# Supplementary figures and images for: Discovery of F-18 labeled repurposed CNS drugs by computational strategy for effective tau imaging and alzheimer’s diagnosis
Source: PLoS One. 2025 Dec 22;20(12):e0338901. doi: 10.1371/journal.pone.0338901 (PMC12721517; doi:10.1371/journal.pone.0338901)

a)

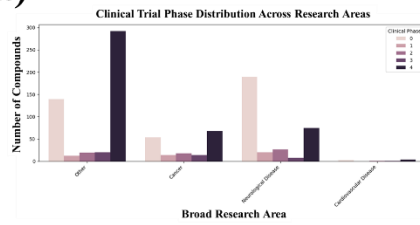

b)

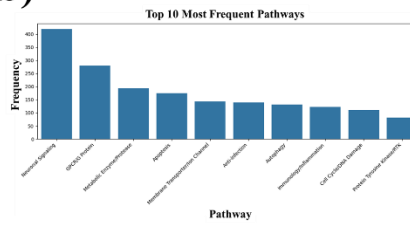

c)

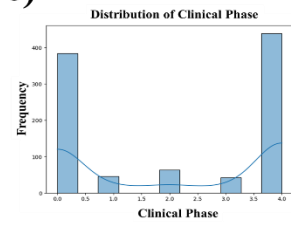

d)

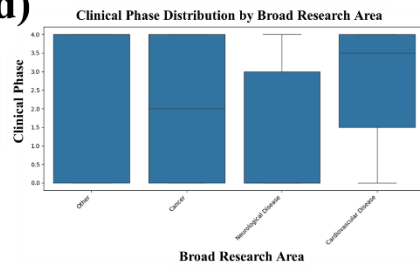

e)

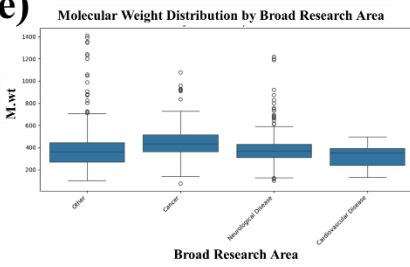

f)

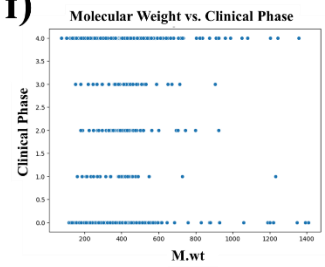

g)

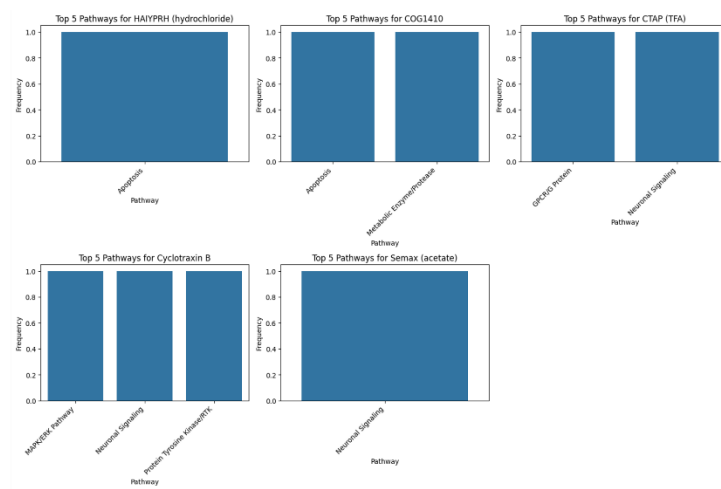

Supplement: S1 Fig — b) Top 10 most frequent biological pathways targeted by the compounds, with notable enrichment in apoptosis, autophagy, and PI3K/Akt/mTOR signaling. c) Overall distribution of clinical phases among the drug candidates, showing a concentration in early (Phase 1–2) and investigational stages. d) Clinical phase distribution stratified by therapeutic research area, showing that oncology-dedicated compounds dominate advanced clinical phases. e) Boxplot of molecular weight (M. Wt) distributions across research areas, demonstrating consistency around the drug-like space (~300–500 Da), with cancer-focused compounds exhibiting slightly broader variability. f) Scatterplot correlating molecular weight with clinical phase, indicating no strong relationship but suggesting a tendency for lower-weight molecules to advance into later phases. g) Top 3 pathways associated with selected key drugs (SKF-96365, CG-806, CUDC-907, Cyclin-Dependent Kinase Inhibitors, and Semaxanib) show target diversity and relevance to neurodegeneration-related mechanisms. (PDF) [file pone.0338901.s001.pdf]

a)

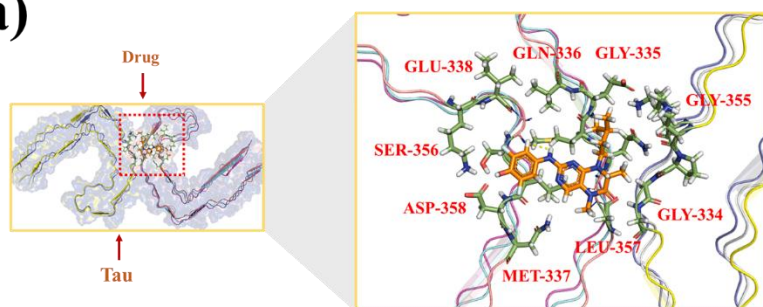

Tau-Drug (388) Complex

Pose 2 ( $\Delta G = -8.10$ )

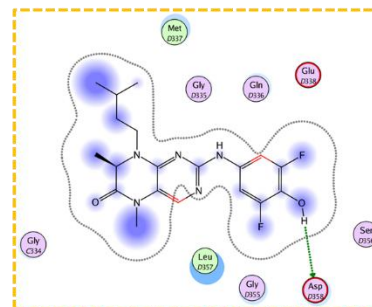

b)

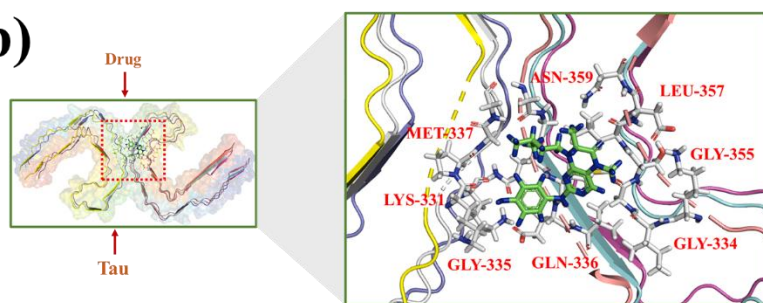

Tau-Drug (388) Complex

Pose 3 ( $\Delta G = -7.85$ )

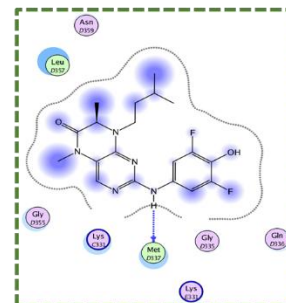

Supplement: S2 Fig — a) 2D & 3D interaction diagram of the 2nd pose of the 388-drug within the active binding pocket of the Tau receptor. b) 2D & 3D interaction diagram of the 3rd pose of the 388-drug within the active binding pocket of the Tau protein. These diagrams offer in-depth perspectives of the molecular interactions, showing the important amino acids that contribute to the binding affinity as well as how the medication attaches to the tau protein. (PDF) [file pone.0338901.s002.pdf]

a)

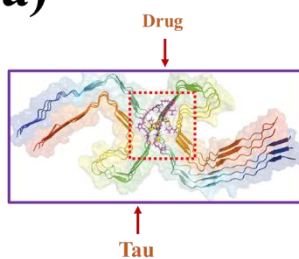

Tau-Drug (416) Complex

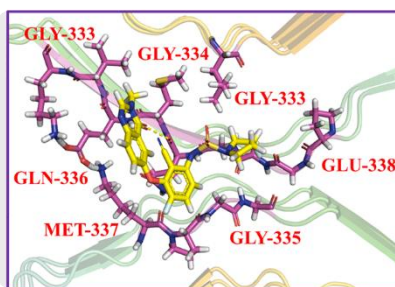

Pose 2 ( $\Delta G = -7.73$ )

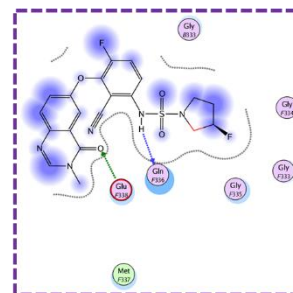

b)

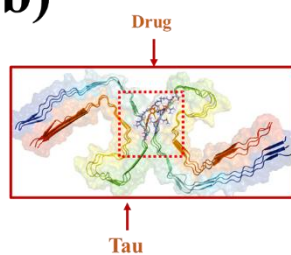

Tau-Drug (416) Complex

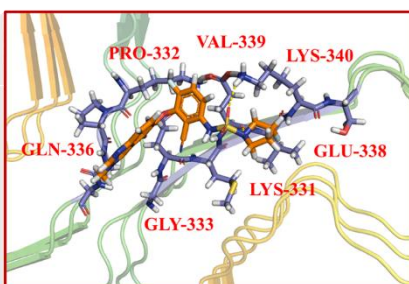

Pose 3 ( $\Delta G = -7.25$ )

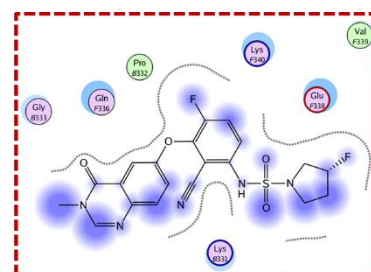

Supplement: S3 Fig — a) 2D & 3D interaction diagram of the 2nd pose of the 416-drug within the active binding pocket of the Tau receptor. b) 2D & 3D interaction diagram of the 3rd pose of the 416-drug within the active binding pocket of the Tau protein. Evaluating the drug’s potential for addressing tau-related neurodegenerative illnesses requires knowledge of the molecular underpinnings of the drug-tau interactions, including the potency of these interactions and important binding residues. (PDF) [file pone.0338901.s003.pdf]

a)

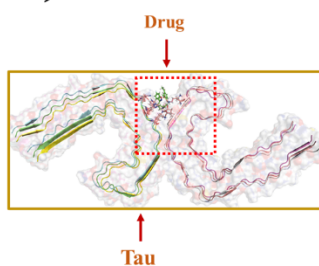

Tau-Drug (610) Complex

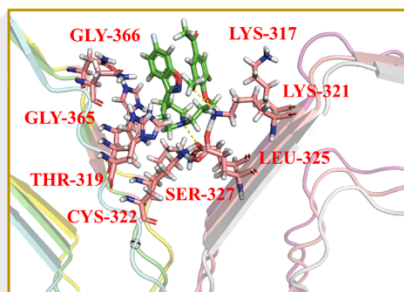

Pose 2 ( $\Delta G = -6.50$ )

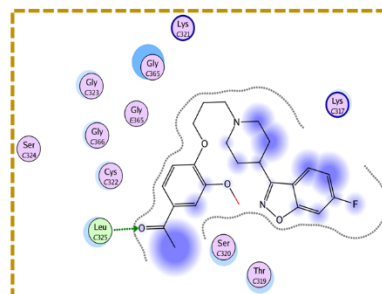

b)

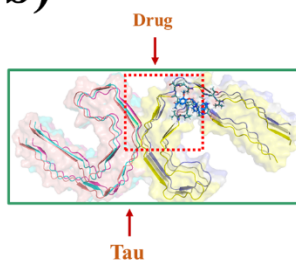

Tau-Drug (610) Complex

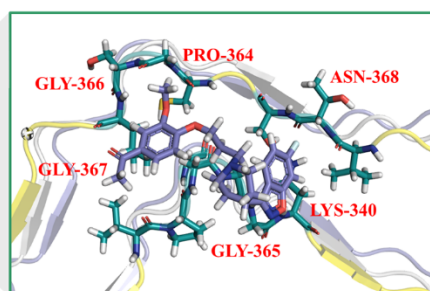

Pose 3 ( $\Delta G = -6.29$ )

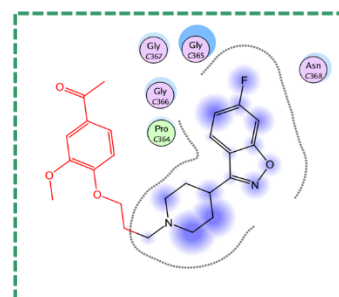

Supplement: S4 Fig — a) 2D & 3D interaction diagram of the 2nd pose of the 416-drug within the active binding pocket of the Tau receptor. b) 2D & 3D interaction diagram of the 3rd pose of the 416-drug within the active binding pocket of the Tau protein. (PDF) [file pone.0338901.s004.pdf]

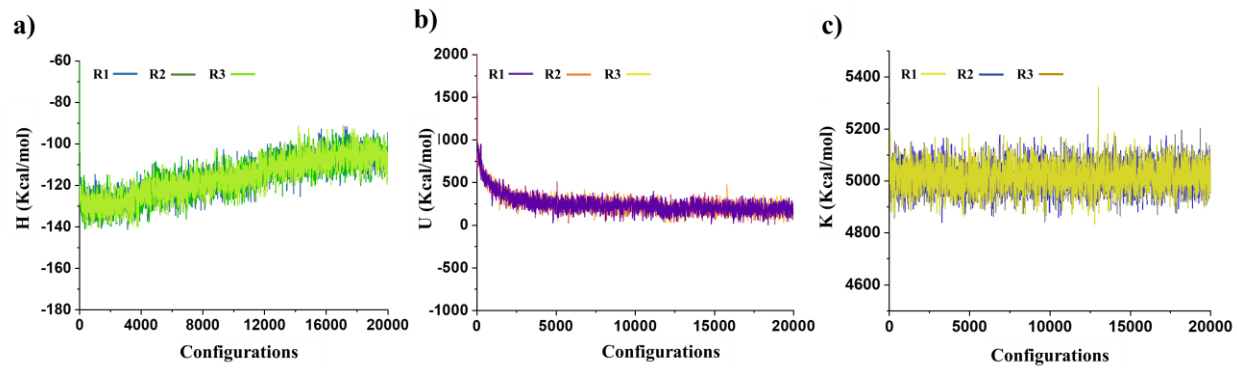

Supplement: S5 Fig — The MD analysis indicates that the system reaches equilibrium and stays constant during the simulation since the total potential energy (H) and kinetic energy (K) are essentially constant. (PDF) [file pone.0338901.s005.pdf]

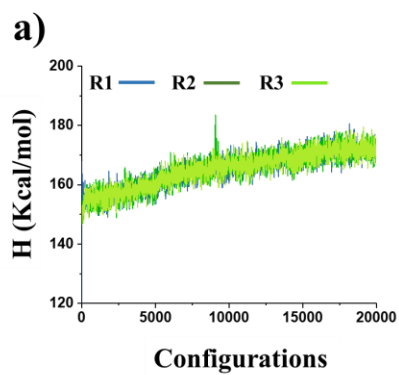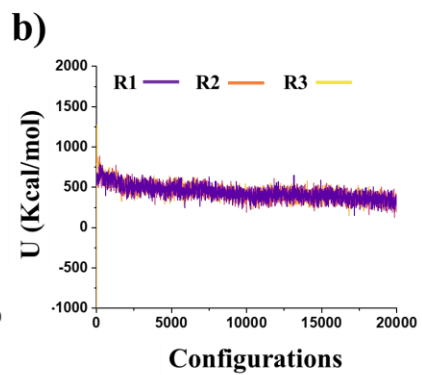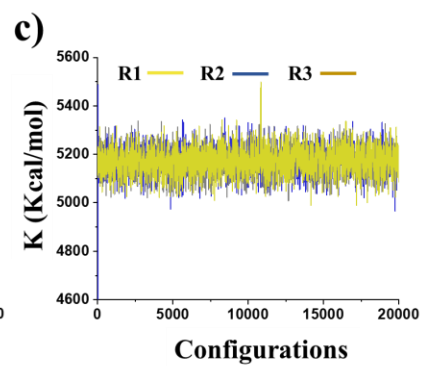

Supplement: S6 Fig — The MD study shows that the total internal energy (U) and the kinetic energy (K) are mostly stable, implying that the system achieves stability and remains constant throughout the experiment. However, the variations in potential energy (H) indicate that the system is adapting when 416 binds to Tau. (PDF) [file pone.0338901.s006.pdf]

**a)**

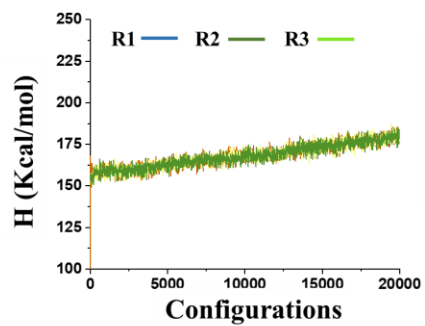

**b)**

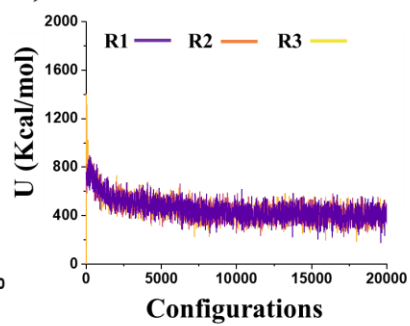

**c)**

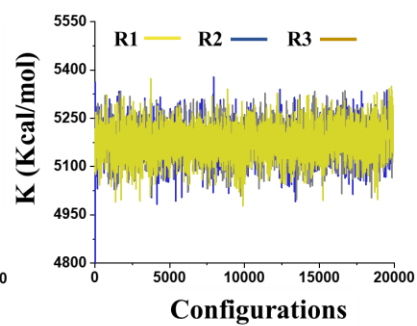

Supplement: S7 Fig — The MD evaluation shows that the total potential energy (H) and the kinetic energy (K) are virtually stable, implying that the system has reached equilibrium and will remain constant across the experiment. However, variations in internal energy (U) indicate that the system is adapting when CBD binds to Tau. (PDF) [file pone.0338901.s007.pdf]
