# Supplementary material for: Discovery of F-18 labeled repurposed CNS drugs by computational strategy for effective tau imaging and alzheimer’s diagnosis
Source: PLoS One. 2025 Dec 22;20(12):e0338901. doi: 10.1371/journal.pone.0338901 (PMC12721517; doi:10.1371/journal.pone.0338901)
Supplement: S1 Table — All pairs show significant differences, with RMSD increasing from drug 318 (tightest binding) to 610 (most flexible ligand). (PDF) [file pone.0338901.s008.pdf]

**Group 1**	**Group 2**	**Mean Diff (Å)**	**Lower CI (Å)**	**Upper CI (Å)**	**p-adj**	**Reject H_0**
318	416	0.653	0.636	0.671	<0.001	Yes
318	610	2.076	2.058	2.094	<0.001	Yes
416	610	1.422	1.405	1.440	<0.001	Yes
