# Supplementary material for: Discovery of F-18 labeled repurposed CNS drugs by computational strategy for effective tau imaging and alzheimer’s diagnosis
Source: PLoS One. 2025 Dec 22;20(12):e0338901. doi: 10.1371/journal.pone.0338901 (PMC12721517; doi:10.1371/journal.pone.0338901)
Supplement: S2 Table — All pairs differ significantly, with drug 416 showing the most stable pocket, while 318 and 610 exhibit greater dynamics (610 > 318). (PDF) [file pone.0338901.s009.pdf]

**Group 1**	**Group 2**	**Mean Diff (Å)**	**Lower CI (Å)**	**Upper CI (Å)**	**p-adj**	**Reject H_0**
318	416	-0.905	-0.917	-0.892	<0.001	Yes
318	610	0.488	0.476	0.501	<0.001	Yes
416	610	1.393	1.380	1.405	<0.001	Yes
