# Supplementary material for: Discovery of F-18 labeled repurposed CNS drugs by computational strategy for effective tau imaging and alzheimer’s diagnosis
Source: PLoS One. 2025 Dec 22;20(12):e0338901. doi: 10.1371/journal.pone.0338901 (PMC12721517; doi:10.1371/journal.pone.0338901)
Supplement: S3 Table — All pairs are significantly different, with drug 318 showing the most protein backbone flexibility, followed by 416 and 610 (most rigid). (PDF) [file pone.0338901.s010.pdf]

**Group 1**	**Group 2**	**Mean Diff (Å)**	**Lower CI (Å)**	**Upper CI (Å)**	**p-adj**	**Reject H_0**
318	416	-4.584	-4.650	-4.517	<0.001	Yes
318	610	-5.483	-5.550	-5.416	<0.001	Yes
416	610	-0.899	-0.965	-0.833	<0.001	Yes
