# Supplementary material for: Discovery of F-18 labeled repurposed CNS drugs by computational strategy for effective tau imaging and alzheimer’s diagnosis
Source: PLoS One. 2025 Dec 22;20(12):e0338901. doi: 10.1371/journal.pone.0338901 (PMC12721517; doi:10.1371/journal.pone.0338901)
Supplement: S4 Table — (PDF) [file pone.0338901.s011.pdf]

**RMSD Metric**	**Drug 318**	**Drug 416**	**Drug 610**
Ligand	0.895 ± 0.143	1.548 ± 0.122	2.970 ± 0.153
Binding Pocket	2.094 ± 0.061	1.189 ± 0.128	2.582 ± 0.088
Protein	10.543 ± 0.569	5.959 ± 0.528	5.060 ± 0.464
