# Supplementary material for: Discovery of F-18 labeled repurposed CNS drugs by computational strategy for effective tau imaging and alzheimer’s diagnosis
Source: PLoS One. 2025 Dec 22;20(12):e0338901. doi: 10.1371/journal.pone.0338901 (PMC12721517; doi:10.1371/journal.pone.0338901)
Supplement: Attachment — Submitted filename: PONE-D-25-38504_RA.pdf [file pone.0338901.s012.pdf]

PLOS ONE

Discovery of F-18 Labeled Repurposed CNS Drugs by Computational Strategy for Effective Tau Imaging and Alzheimer's Diagnosis

--Manuscript Draft--

**Manuscript Number:**	PONE-D-25-38504
**Article Type:**	Research Article
**Full Title:**	Discovery of F-18 Labeled Repurposed CNS Drugs by Computational Strategy for Effective Tau Imaging and Alzheimer's Diagnosis
**Short Title:**	Discovery of F-18 Labeled Repurposed CNS Drugs for A Alzheimer's
**Corresponding Author:**	Chong Cheng<br>Central South University Xiangya School of Medicine<br>Changde city, CHINA
**Keywords:**	Alzheimer's disease (AD), Tau protein imaging, F-18 labeled drug candidates, PET imaging for Alzheimer's diagnosis, Blood-brain barrier (BBB) permeability
**Abstract:**	Alzheimer’s disease (AD) remains a significant challenge in diagnosis and treatment, with current methods insufficient for early detection. A major obstacle is the lack of effective imaging agents targeting the Tau protein, which plays a key role in AD pathology. To address this, we developed a computational methodology for selecting F-18 labeled drug candidates from a library of CNS-penetrant compounds curated from literature and databases. The library, consisting of 977 compounds, was evaluated based on clinical data, target proteins, pathways, toxicity, and other relevant factors. We implemented Python-based algorithms to select the top 39 compounds from virtual screening results, prioritizing those with optimal Tau binding affinity and BBB permeability. Additionally, we developed an algorithm to identify F-18 labeling candidates that maintain their biological activity post-labeling. We then performed virtual screening of an F-18 labeled drug library and selected the top 3 compounds based on stability and imaging potential. The selected compounds exhibited molecular weights of 350 to 520 Da, favorable logP values (2.05-2.72), and high BBB permeability. Our results show that the drug 388 (BI-D1870) is an ATP-competitive) binds to Tau with binding free energies of ΔG = -8.79 kcal/mol, drug 416 (BRAF inhibitor, RG6344) have binding free energy of ΔG = -7.91 kcal/mol and the drug 610 (Drug 610 (Iloperidone/HP 873)) is a D2/5-HT2 receptor antagonist) having the predicted binding free energy of ΔG = -6.88 kcal/mol respectively. Molecular dynamics simulations confirmed stable Tau-drugs interactions, with minimal RMSD fluctuations, indicating strong binding. The F-18 label enabled real-time PET imaging, allowing non-invasive tracking of the drug’s binding to Tau in the brain. Our approach provides a comprehensive solution to the current limitations in Alzheimer’s diagnosis by offering F-18 labeled drugs that effectively target Tau protein without compromising their biological activity, advancing both diagnostic and therapeutic strategies for AD.
**Order of Authors:**	Pan Tang
	Xuehua Chen
	Pingping Li
	Ling Zhang
	Bin Tang
	Jun Wen
	Yan Liu
	Iqra Kalsoom
	Chong Cheng
**Additional Information:**	
**Question**	**Response**

Powered by Editorial Manager® and ProduXion Manager® from Aries Systems Corporation

**Financial Disclosure**<br><br>Enter a financial disclosure statement that describes the sources of funding for the work included in this submission. Review the submission guidelines for detailed requirements. View published research articles from *PLOS ONE* for specific examples.<br><br>This statement is required for submission and **will appear in the published article** if the submission is accepted. Please make sure it is accurate.<br><br>**Funded studies**<br>Enter a statement with the following details:<br>• Initials of the authors who received each award<br>• Grant numbers awarded to each author<br>• The full name of each funder<br>• URL of each funder website<br>• Did the sponsors or funders play any role in the study design, data collection and analysis, decision to publish, or preparation of the manuscript?<br><br>Did you receive funding for this work?	Yes
Please add funding details.<br>as follow-up to "**Financial Disclosure**<br><br>Enter a financial disclosure statement that describes the sources of funding for the work included in this submission. Review the submission guidelines for detailed requirements. View published research articles from *PLOS ONE* for specific examples.<br><br>This statement is required for submission and **will appear in the published article** if the submission is accepted. Please make sure it is accurate.	This work was supported by an active grant from the Changde Science and Technology Bureau (Technology R&D and Innovation Guidance Program; Grant/Award Number: Changke Han [2022] No. 51) with project title "Application of Modified Volume-Viscosity Swallow Test in Neurodegenerative Diseases".

Powered by Editorial Manager® and ProduXion Manager® from Aries Systems Corporation

**Funded studies**<br>Enter a statement with the following details:<br>• Initials of the authors who received each award<br>• Grant numbers awarded to each author<br>• The full name of each funder<br>• URL of each funder website<br>• Did the sponsors or funders play any role in the study design, data collection and analysis, decision to publish, or preparation of the manuscript?<br><br>Did you receive funding for this work?"	
Please select the country of your main research funder (please select carefully as in some cases this is used in fee calculation).<br>as follow-up to "**Financial Disclosure**<br><br>Enter a financial disclosure statement that describes the sources of funding for the work included in this submission. Review the submission guidelines for detailed requirements. View published research articles from *PLOS ONE* for specific examples.<br><br>This statement is required for submission and **will appear in the published article** if the submission is accepted. Please make sure it is accurate.<br><br>**Funded studies**<br>Enter a statement with the following details:<br>• Initials of the authors who received each award<br>• Grant numbers awarded to each author<br>• The full name of each funder<br>• URL of each funder website<br>• Did the sponsors or funders play any role in the study design, data collection and analysis, decision to publish, or preparation of the manuscript?<br><br>Did you receive funding for this work?"	CHINA - CN
**Competing Interests**	The authors have declared that no competing interests exist.

Use the instructions below to enter a competing interest statement for this submission. On behalf of all authors, disclose any competing interests that could be perceived to bias this work—acknowledging all financial support and any other relevant financial or non-financial competing interests.<br><br>This statement is required for submission and **will appear in the published article** if the submission is accepted. Please make sure it is accurate and that any funding sources listed in your Funding Information later in the submission form are also declared in your Financial Disclosure statement.<br><br>View published research articles from *PLOS ONE* for specific examples.<br><br>**NO authors have competing interests**<br><br>Enter: *The authors have declared that no competing interests exist.*<br><br>**Authors with competing interests**<br><br>Enter competing interest details beginning with this statement:<br><br>*I have read the journal's policy and the authors of this manuscript have the following competing interests: [insert competing interests here]*<br><br>* typeset	
**Ethics Statement**<br><br>Enter an ethics statement for this submission. This statement is required if the study involved:<br><br>• Human participants<br>• Human specimens or tissue<br>• Vertebrate animals or cephalopods<br>• Vertebrate embryos or tissues	"N/A"

Powered by Editorial Manager® and ProduXion Manager® from Aries Systems Corporation

• Field research<br><br>Write "N/A" if the submission does not require an ethics statement.<br><br>General guidance is provided below. Consult the submission guidelines for detailed instructions. **Make sure that all information entered here is included in the Methods section of the manuscript.**	

Powered by Editorial Manager® and ProduXion Manager® from Aries Systems Corporation

**Format for specific study types**<br><br>**Human Subject Research (involving human participants and/or tissue)**<br>• Give the name of the institutional review board or ethics committee that approved the study<br>• Include the approval number and/or a statement indicating approval of this research<br>• Indicate the form of consent obtained (written/oral) or the reason that consent was not obtained (e.g. the data were analyzed anonymously)<br><br>**Animal Research (involving vertebrate animals, embryos or tissues)**<br>• Provide the name of the Institutional Animal Care and Use Committee (IACUC) or other relevant ethics board that reviewed the study protocol, and indicate whether they approved this research or granted a formal waiver of ethical approval<br>• Include an approval number if one was obtained<br>• If the study involved *non-human primates*, add *additional details* about animal welfare and steps taken to ameliorate suffering<br>• If anesthesia, euthanasia, or any kind of animal sacrifice is part of the study, include briefly which substances and/or methods were applied<br><br>**Field Research**<br><br>Include the following details if this study involves the collection of plant, animal, or other materials from a natural setting:<br>• Field permit number<br>• Name of the institution or relevant body that granted permission	
**Data Availability**<br><br>Authors are required to make all data underlying the findings described fully available, without restriction, and from the time of publication. PLOS allows rare exceptions to address legal and ethical concerns. See the PLOS Data Policy and FAQ for detailed information.	Yes - all data are fully available without restriction

A Data Availability Statement describing where the data can be found is required at submission. Your answers to this question constitute the Data Availability Statement and **will be published in the article**, if accepted.<br><br>**Important:** Stating 'data available on request from the author' is not sufficient. If your data are only available upon request, select 'No' for the first question and explain your exceptional situation in the text box.<br><br>Do the authors confirm that all data underlying the findings described in their manuscript are fully available without restriction?	
**Describe where the data may be found in full sentences. If you are copying our sample text, replace any instances of XXX with the appropriate details.**<br><br>• If the data are **held or will be held in a public repository**, include URLs, accession numbers or DOIs. If this information will only be available after acceptance, indicate this by ticking the box below. For example: *All XXX files are available from the XXX database (accession number(s) XXX, XXX.*).<br>• If the data are all contained **within the manuscript and/or Supporting Information files**, enter the following: *All relevant data are within the manuscript and its Supporting Information files.*<br>• If neither of these applies but you are able to provide **details of access elsewhere**, with or without limitations, please do so. For example:<br><br>*Data cannot be shared publicly because of [XXX]. Data are available from the XXX Institutional Data Access / Ethics Committee (contact via XXX) for researchers who meet the criteria for access to confidential data.*<br><br>*The data underlying the results presented in the study are available from (include the name of the third party*	All relevant data are within the manuscript and its Supporting Information files.

and contact information or URL).<br>• This text is appropriate if the data are owned by a third party and authors do not have permission to share the data.<br><br>* typeset	
Additional data availability information:	Tick here if the URLs/accession numbers/DOIs will be available only after acceptance of the manuscript for publication so that we can ensure their inclusion before publication.

Manuscript Click here to access/download;Manuscript;Final Manuscript.docx

Discovery of F-18 Labeled Repurposed CNS Drugs by Computational Strategy for Effective Tau Imaging and Alzheimer's Diagnosis

Pan Tang[1], Xuehua Chen[1], Pingping Li[2], Ling Zhang[3], Bin Tang[3], Jun Wen[1], Yan Liu[1], Iqra Kalsoom[4], Chong Cheng[1]*

[1]**Department of Neurology & Department of Nuclear Medicine**, Changde Hospital, Xiangya School of Medicine, Central South University (The First People's Hospital of Changde), Changde 415000, China
[2]**Department of Paediatrics**, Changde Hospital, Xiangya School of Medicine, Central South University (The First People's Hospital of Changde), Changde 415000, China
[3]**Centre for Reproductive Medicine**, Changde Hospital, Xiangya School of Medicine, Central South University (The First People's Hospital of Changde), Changde 415000, China
[4]**School of Chemistry and Chemical Engineering,** Beijing Institute of Technology, Beijing 102401, China
*__Corresponding author__: Chong Cheng (j001cg@163.com)

Abstract

Alzheimer's disease (AD) remains a significant challenge in diagnosis and treatment, with current methods insufficient for early detection. A major obstacle is the lack of effective imaging agents targeting the Tau protein, which plays a key role in AD pathology. To address this, we developed a computational methodology for selecting F-18 labeled drug candidates from a library of CNS-penetrant compounds curated from literature and databases. The library, consisting of 977 compounds, was evaluated based on clinical data, target proteins, pathways, toxicity, and other relevant factors. We implemented Python-based algorithms to select the top 39 compounds from virtual screening results, prioritizing those with optimal Tau binding affinity and BBB permeability. Additionally, we developed an algorithm to identify F-18 labeling candidates that maintain their biological activity post-labeling. We then performed virtual screening of an F-18 labeled drug library and selected the top 3 compounds based on stability and imaging potential. The selected compounds exhibited molecular weights of 350 to 520 Da, favorable logP values (2.05-2.72), and high BBB permeability. Our results show that the drug 388 (BI-D1870) is an ATP-competitive) binds to Tau with binding free energies of ΔG = -8.79 kcal/mol, drug 416 (BRAF inhibitor, RG6344) have binding free energy of ΔG = -7.91 kcal/mol and the drug 610 (Drug 610 (Iloperidone/HP 873)) is a D2/5-HT2 receptor antagonist) having the predicted binding free energy of ΔG = -6.88 kcal/mol respectively. Molecular dynamics simulations confirmed stable Tau-drugs interactions, with minimal RMSD fluctuations, indicating strong binding. The F-18

label enabled real-time PET imaging, allowing non-invasive tracking of the drug’s binding to Tau in the brain. Our approach provides a comprehensive solution to the current limitations in Alzheimer’s diagnosis by offering F-18 labeled drugs that effectively target Tau protein without compromising their biological activity, advancing both diagnostic and therapeutic strategies for AD.

Key Words: Alzheimer’s disease (AD)**,** Tau protein imaging**,** F-18 labeled drug candidates**,** PET imaging for Alzheimer’s diagnosis**,** Blood-brain barrier (BBB) permeability

Introduction

Alzheimer’s disease (AD) stands as the predominant form of dementia, representing a global health challenge with significant clinical and societal impact [1]. It is a progressive neurodegenerative disorder that impacts cognition function as well as behaviour [2] . According to the World Health Organization (WHO), over 55 million people worldwide suffer from AD or other types of dementia, and every year there are nearly 10 million new cases [3]. In the United States, about one out of nine individuals (10.8%) age 65 and older have AD, with occurrence of 1275 new cases per 100,000 person annually [4]. There is yet no cure for AD unfortunately and patients are usually diagnosed at a late stage with an average survival period of 4-8 years [1, 5].

Pathologically, Alzheimer's disease is a progressive disorder characterized by the deposition of extracellular plaques composed of aggregated forms of the amyloid-beta (Aβ) polypeptide and intraneuronal neurofibrillary tangles (NFTs) composed of aggregated hyperphosphorylated tau protein [6]. Tau pathology is marked to correlate more with neuronal loss, neurodegeneration and disease progression , making it a critical target for disease staging and therapeutic monitoring [7]. As Early and accurate diagnosis in AD remains a challenge, Nuclear medicine offers a powerful tool offering valuable insights into disease progression and diagnosis [8, 9]. Several radiopharmaceuticals are employed in advanced imaging techniques, such as positron emission tomography (PET) and single-photon emission computed tomography (SPECT), to enable non-invasive and real-time visualization of the molecular processes [10-12].

Nuclear medicine is experiencing a radical shift with the emergence of theragnostic by integrating diagnostic imaging with targeted therapy [13, 14]. Several Aβ- and tau-targeting radiotracers such

as [^{18}F]florbetapir (Amyvid®), [^{18}F]florbetaben (Neuraceq®), [^{18}F]flutemetamol (Vizamyl®), and [^{18}F]T807 (Tauvid®) have received FDA approval for PET imaging in patients with cognitive impairment [15, 16]. Imaging biomarkers have demonstrated clinical utility but also have some certain limitations [17]. Some tracers have shown off target binding to monoamine oxidase enzymes and melanin containing region , limited CNS penetration , slow elimination rates , nonspecific uptakes that reduces diagnostic clarity (McKhann et al., 2011; Wong et al., 2021) [18].

In CNS drug discovery, the assessment of brain exposure is critical for lead optimization as the compounds need to cross the blood-brain barrier (BBB) to reach their therapeutic targets [19]. The BBB is a complex system involving passive and active mechanisms of transport and efflux transporters [20, 21]. Several PET tracers have been developed that are tau specific but most of them have certain limitations like poor CNS penetration, nonspecific binding, inadequate selectivity for pathological tau over other proteins [22]. The heterogeneity and complex structure of tau aggregates in AD necessitates highly specific and bioavailable compounds with well-defined pharmacokinetic profile [23]. Hence , using CNS penetrant compound library and evaluating the physicochemical properties like molecular weights ,we aim to evaluate existing shortcomings in this regard [24]. In addition to it, the development of a florine-18 labelled compound from CNS penetrant chemical library offers target specificity, high positron yield , favourable half-life and compatibility with radiochemistry [25].

In this context , we are persuading to extract a library of CNS penetrant compounds that could be radio labelled using Florine-18 radionucleotide [26]. In silico studies for the potential candidates from library and their virtual screening are performed, allowing efficient prioritization of molecules with better blood brain bioavailability [27-29]. In addition Molecular modelling and molecular docking studies were performed with Subsequent ADMET predictions that provide pharmacokinetic profiles, including blood-brain barrier permeability, metabolic stability, and safety parameters [30]. Our findings demonstrate that F-18 labelled drugs, 388 (BI-D1870) binds with Tau protein with binding free energies of ΔG = -8.79 kcal/mol, drug 416 BRAF inhibitor/RG6344 have binding free energy of ΔG = -7.91 kcal/mol and the 610 (Iloperidone/HP 873) having the predicted binding free energy of ΔG = -6.88 kcal/mol respectively. By integrating CNS drug-likeness parameters with radiolabeling feasibility, this study seeks to prioritize

compounds with high translational potential for development as diagnostic agents in nuclear medicine, thereby accelerating the preclinical screening process and supporting the advancement of novel imaging probes for Alzheimer's disease.

Materials and Methods

This study employed an integrated computational pipeline to identify and optimize CNS-penetrant compounds for tau-targeted AD diagnostics and therapeutics. Virtual screening of a curated library of blood-brain barrier (BBB)-permeable drugs (SDF format) was conducted using MOE (Molecular Operating Environment), with molecular docking against the tau protein (PDB: 1T28). Compounds were preprocessed for physiological pH (7.4) and evaluated for drug-likeness (LogP: 2.5-3.8; MW: 350-520 Da). Top hits were assessed for 18F-radiolabeling feasibility via a custom RDKit script, which identified fluorine sites for isotopic substitution. ADMET properties (e.g., BBB penetration, solubility) were predicted using MOE and SwissADME. Finally, molecular dynamics (MD) simulations (100 ns, Amber99sb force field) analyzed binding stability for prioritized 18F18F-labeled candidates. This workflow synergized structure-based screening, radiolabeling prediction, and dynamic validation to advance tau-directed PET probes and therapeutics.

Retrieving BBB Permeable Drugs Library and Virtual Screening Against Tau Protein

To construct an initial virtual screening library for blood-brain barrier (BBB)-penetrant drugs targeting Tau protein, we conducted a structured literature mining using PubMed. The search employed key terms such as "blood-brain barrier crossing drugs", "clinically tested drugs", and "drugs with known targets and pathways". From the results, 977 compounds were selected based on established or predicted ability to cross the BBB, documented clinical development status, and well-characterized molecular targets and pathways. The compounds were curated to ensure relevance in neurological applications and potential imaging adaptability, particularly for PET. Each selected drug was annotated with information including molecular weight, SMILES structure, target classes (e.g., mTOR, CDK, autophagy modulators), biological activity, and clinical phase status. The library was designed with the intent to identify 18F-labelable candidates for subsequent PET radiotracer development (Figure S1). Moreover, a virtual screening approach was implemented to identify potential candidates for diagnostic imaging and therapeutic treatment

of AD. The screening was performed on curated library of CNS-penetrant drugs, using MOE (Molecular Operating Environment) for molecular docking. The drug database included substances with recognized blood-brain barrier (BBB) penetration properties and was supplied in SDF format. MOE's structure preparations tools were used to create and validate the SDF file that contained a library of CNS medications. To make that the right ionization modes were allocated for docking simulations, the compounds were hydrogenated at physiological pH (7.4). After obtaining the Tau protein structure via the Protein Data Bank (PDB) (PDB ID: 9EOH), MOE's energy-minimizing methodology was used to refine the structure and prepare it for docking. By removing any steric conflicts or unfavorable bond geometries from the structure, this step guaranteed the stability of the target protein. The molecular weights ranged from 350 to 520 Da, that is the optimal range for compounds that resemble drugs. Values for solubility fell within acceptable bounds for intravenous delivery. All of the chosen drugs showed appropriate LogP values (between 2 and 4), confirming high blood-brain barrier (BBB) penetration.

Radiolabeling Prediction for F-18 Labeled Compounds

The next step after the virtual screening was to determine whether fluorine-18 (F-18) labeling for imaging using PET was feasible. Since F-18 is frequently utilized in PET scans because of its advantageous qualities, the F-18 labeling procedure was carried out by substituting Fluorine (F) atoms in the discovered high-affinity molecules with Fluorine-18 (F-18). To find the functional groups in the evaluated compounds that included fluorine, a custom Python script was created using RDKit. This script evaluated each chemical for fluorine (F) atoms and projected the possibility of labeling with fluorine-18 by switching the F atoms with F-18. The substances with the most effective labeling positions were identified as candidates for PET imaging. This script evaluated the SDF database of top docking hits, produced new SDF files with the labeled compounds, and exposed them to a further round of virtual screening.

Molecular Docking Studies

Molecular docking has become an important method in the recognition and creation of lead compounds, offering a quick and economical alternative to laboratory analysis. Virtual screening, which examines the binding interactions and processes of multiple compounds, is critical to this technology. The desired Tau protein and compound library was docked employing the MOE docking module. The atoms of hydrogen were incorporated to the target protein structure, and

energy minimization was performed using MOPAC7.0. The ligands were docked in flexible mode, whereas the receptor (Tau) remained rigid. Docking calculations were run to determine each compound's binding potential using MOE's affinity scoring tool. The docking data were ordered by binding energy, and the leading candidates were chosen for their favourable binding interactions with the Tau binding site.

ADMET and Drug-likeness Prediction of the Target F-18 Labeled Drugs

Drug-likeness screening is required to identify potential therapeutic compounds in the early stages of drug development. Identifying the chemicals' ADMET (Absorption, Distribution, Metabolism, Excretion, and Toxicity) characteristics is a key step in the process. To determine the efficacy of these F-18 tagged compounds for in vivo application, ADMET parameters were anticipated. The properties of logP (lipophilicity), the molecular weight, the solubility, and BBB permeability were estimated utilizing MOE and RDKit. The above characteristics were utilized to determine that the selected drugs had good CNS penetration and could be useful choices for PET imaging and therapy. For additional confirmation of the validity of pharmacological ADMET analysis, researchers can use the simple SwissADME platform (http://www.swissadme.ch/) to assess the possible physiological consequences of therapeutic pharmaceuticals.

Molecular Dynamics Simulations for Tau Binding

The binding strength and dynamics of the leading three F-18 labeled candidate drugs, including 388 (BI-D1870), drug 416 (BRAF inhibitor/RG6344), and drug 610 (Iloperidone/HP 873), were evaluated using molecular dynamics (MD) models. The simulation process was conducted using MOE with the Amber99sb force field. MD simulations were run for a period of 100 ns to evaluate the stability of the drug-Tau complex over time. The top three F-18 labeled compounds were first docked into the Tau binding pocket and the resulting protein-ligand complex was solvated using the TIP3P water model. Counterions were added to neutralize the system. Energy minimization was performed to remove any steric clashes, followed by NVT (constant volume and temperature) and NPT (constant pressure and temperature) equilibration. The MD simulations were run at 300 K temperature and 1 bar pressure for 100 ns. The systems were monitored for RMSD (Root Mean Square Deviation) analysis to evaluate the stability of both the ligand and protein complexes during the simulation.

Results and Discussion

The final retrieved and assembled library comprised 977 BBB-penetrant drug candidates with diverse mechanisms of action relevant to neurodegeneration and Tau pathology, including kinase inhibitors (e.g., CDK and mTOR inhibitors), autophagy regulators, and apoptosis inducers. Notable examples include Flavopiridol (Phase 2, CDK inhibitor), PQR620 (mTOR inhibitor), and SNS-032 (CDK inhibitor, Phase 1). Each compound was assessed for molecular weight, biological targets, and clinical phase data, alongside SMILES representations to facilitate in silico screening. Based on their pharmacological profile, known safety data, and chemical structures amenable to F-18 substitution, these compounds serve as a rationally designed set for docking studies and radiolabeling potential. The next step will involve computational binding affinity evaluation with Tau protein, followed by selection of the top three candidates for F-18 labeling and PET imaging feasibility studies.

Virtual Screening Results for Tau

After conducting the virtual screening of 977 compounds using MOE, 39 compounds from the CNS-penetrant drug library were identified as strong binders to Tau (Figure 1). These compounds exhibited binding affinities with Tau ranging from -6.00 to -9.0 kcal/mol, demonstrating favorable interactions with the target protein. Specifically, the top 10 drug candidates had hydrophobic interactions and hydrogen bonds with the Tau microtubule-binding region, which is crucial for maintaining Tau's function and stability. These 39 compounds were successfully modeled with ^{18}F atoms incorporated at viable positions, preserving chemical stability and pharmacophore integrity. Subsequent ADMET profiling further refined the selection of three compounds with molecular weights between 425 – 459 Da, within the acceptable drug-like range (350–520 Da), and demonstrated favorable solubility for intravenous delivery. Most importantly, logP values ranged from 2.05 to 2.72, indicating high BBB permeability, a critical parameter for neuroimaging agents. As visualized in Figure S1 a (clinical phase vs. research area) and e/f (molecular weight vs. clinical phase), these three candidates also aligned well with existing drug development trends and CNS-penetrant physicochemical space. Together, their strong Tau affinity, labeling feasibility, favorable ADMET properties, and CNS compatibility support their prioritization for future PET probe development in neurodegenerative disease research.

Current Status of Selected Drugs

The selected drugs, 388 (BI-D1870), 416 (RG6344), and 610 (Iloperidone) presented in Figure 2, have shown promising potential in crossing the blood-brain barrier (BBB), making them significant candidates in neurological and cancer research. BI-D1870, an ATP-competitive inhibitor of ribosomal S6 kinase [31] isoforms, has demonstrated potent activity with low nanomolar IC50 values for all RSK isoforms, indicating its potential as a therapeutic agent for autophagy and MAPK/ERK pathway-related disorders. However, there is currently no development report available for this drug. RG6344, a potent BRAF inhibitor, is specifically designed for targeting BRAF V600-mutant solid tumors, such as colorectal cancer. It is known for its activity in the MAPK/ERK pathway, but like BI-D1870, no clinical development has been reported so far. Iloperidone, an atypical antipsychotic drug, is already launched and used for the treatment of schizophrenia symptoms. It acts as a dual antagonist for both dopamine and serotonin receptors, contributing to its efficacy in neurological disorders. Despite their varied therapeutic targets, these drugs demonstrate effective blood-brain barrier penetration, which makes them valuable for further exploration in both cancer and neurological disease research. However, it is important to note that while Iloperidone has already been marketed, the other two drugs, BI-D1870 and RG6344, are still in the preclinical or early-stage development phase.

Interaction Analysis of Drugs with Tau Protein

The F-18 labeled drug 388 (BI-D1870) interacts with several key amino acid residues of the Tau protein as shown in Figure 3a**.** The interaction diagram gives a detailed spatial view of how the drug interacts at the molecular level with the Tau protein structure, possibly inhibiting or modulating the Tau protein's role in Alzheimer's disease. The drug likely binds through hydrophobic interactions with the LEU-357, MET-337, PRO-332, GLN-336, GLU-338, LYS-340, GLY-355 and VAL-339, which prefer to interact with nonpolar groups crucial for the drug's action. The presence of alkyl or aromatic groups in the drug molecule likely facilitates these interactions that help stabilize the drug-protein complex. Further, the polar functional groups such as carbonyl or hydroxyl groups of the drug forms a hydrogen bond with LYS-331. The predicted binding free energy of the best three poses of drug-Tau complexes is ΔG = -8.79 kcal/mol, -8.10 kcal/mol and -7.85 kcal/mol respectively. The drug is shown to bind effectively to these residues, forming

critical interactions that is key to the drug's potential role in treating Alzheimer's disease, as it may disrupt Tau aggregation or improve brain function.

The Figure 3b illustrates the binding between an F-18 labeled drug 416 (BRAF inhibitor/RG6344) and the Tau protein, a key player in Alzheimer's disease. The diagram shows that the drug interacts with several specific amino acid residues on the Tau protein, including HIS-329, ASN-327, PRO-332, LYS-331, GLU-338 and LYS-340. These residues play a crucial role in the drug's effectiveness by forming various molecular interactions. Hydrophobic interactions are likely established between the drug and the nonpolar amino acid residues including PRO-332, LYS-331, LYS-340, ASN-327 and HIS-329, helping to anchor the drug within the hydrophobic pocket of the Tau protein. Moreover, the negatively charged GLU-338 residue may engage in electrostatic interactions with positively charged groups on the drug, further enhancing binding affinity. Van der Waals forces also contribute to the interaction, particularly with residues like PRO-332. The predicted binding free energy of the best three poses of drug-Tau complexes is $\Delta G = -7.91$ kcal/mol, -7.73 kcal/mol and -7.25 kcal/mol respectively. The F-18 isotope attached to the drug 416-BRAF inhibitor/RG6344 plays a significant role in its detection, enabling the drug's distribution and interaction with Tau to be monitored through PET scans.

The binding interaction between F-18 labeled drug 610 (Iloperidone/HP 873) and the Tau protein is shown in Figure 3c. The Drug 610 (Iloperidone/HP 873) interacts with key residues in the Tau protein, including HIS-329, GLU-338, LYS-340, and LYS-331, each contributing to the drug's binding stability. HIS-329 and LYS-331 forms both hydrogen bonds with the drug's polar functional groups, anchoring it to the protein. Additionally, the carboxyl group of GLU-338 participates in electrostatic interactions with the drug's positively charged regions, enhancing the binding affinity. These interactions are crucial for the effective binding of the Drug 610 (Iloperidone/HP 873) to the Tau protein, suggesting its potential as a therapeutic agent for Alzheimer's disease. The predicted binding free energy of the best three poses of drug-Tau complexes is $\Delta G = -6.88$ kcal/mol, -6.50 kcal/mol and -6.29 kcal/mol respectively. The docking score of all the complexes with CBD is presented in Table 1.

Hence, the radiolabeled nature of the drug (F-18) enables its tracking through positron emission tomography (PET) imaging and monitoring the drug's distribution in the body, particularly in the

brain, where Tau pathology is prominent. The radiolabeling with F-18 enhances the ability to visualize and track the drug's behavior in vivo, which is valuable for assessing its effectiveness in clinical settings. This combination of targeted drug binding and the diagnostic capability of F-18 labeling makes the drug an effective candidate for both treating Alzheimer's disease and monitoring treatment efficacy in clinical settings.

Table 1. Docking score and interacting binding residues of Tau protein with different labelled drugs.

Drugs	Receptor	Complex Poses	Binding Free Energy (Kcal/mol)	Amino Acid Residues involved in binding interactions
388 (BI-D1870)	Tau	Pose 1<br>Pose 2<br>Pose 3	-8.79<br>-8.10<br>-7.25	LEU-357, MET-337, PRO-332, GLN-336, GLU-338, LYS-340, GLY-355 and VAL-339
BRAF inhibitor/RG 6344	-	Pose 1<br>Pose 2<br>Pose 3	-7.91<br>-7.73<br>-7.85	HIS-329, ASN-327, PRO-332, LYS-331, GLU-338 and LYS-340
Drug 610 (Drug 610 (Iloperidone/ HP 873)	-	Pose 1<br>Pose 2<br>Pose 3	-6.88<br>-6.50<br>-6.29	HIS-329, GLU-338, LYS-340, and LYS-331

(Pose 2 and 3 of all drugs are presented in S2, S3 and S4)

MD Simulation Analysis

The docked complexes of the top three F-18 labeled drugs (388 (BI-D1870), BRAF inhibitor/RG6344 and Drug 610 (Iloperidone/HP 873) with Tau receptor underwent MD simulations to assess the stability of their protein-ligand complexes. The simulations were conducted over a period of 100 ns. The RMSD analysis of the ligand, binding pocket, and protein in complex with the drug offers valuable insights into the structural stability and dynamics of the system throughout the MD simulation.

RMSD Analysis of Tau with Drug 388 (BI-D1870) Complex

RMSD analysis of the Tau-388 (BI-D1870) complex shows that initially, the ligand has notable fluctuations during the first 1-10 ns, indicating that it is adjusting its conformation within the

binding pocket to find its most stable binding pose (Figure 4a). Then from 10-30 ns, the ligand stabilizes, suggesting that it has found a stable position within the binding pocket, where it interacts favorably with key residues of the protein. After 50 ns, the ligand's RMSD remains stable with minor fluctuations throughout the 100 ns, indicating strong binding and that the ligand is well-anchored in the binding site with no major structural changes.

The protein RMSD reveals that the protein undergoes initial fluctuations as it rearranges after the drug binds, which is normal as the protein adapts to the complex (Figure 4b). The F-18 label, being an isotope, may cause slight variations in the drug's orientation within the Tau binding pocket due to its interactions with neighboring residues. These fluctuations are necessary for the drug to find its most stable conformation that maximizes its interaction with the Tau protein. After a short time, duration, the protein stabilizes as its structure becomes rigidified by the drug's binding, and the interactions with the ligand help to stabilize the overall complex. After 20 ns, the protein's RMSD remains relatively constant throughout the MD, with only minor fluctuations, indicating that the protein's structure remains stable in the drug-bound state for the rest of the simulation.

The binding pocket's RMSD shows considerable fluctuations at the start of the simulation, reflecting the pocket's flexibility as it adapts to accommodate the ligand (Figure 4c). After that, the RMSD decreases and stabilizes, indicating that the pocket has adjusted to the ligand's presence, allowing the protein to adopt a stable conformation. Despite this stabilization, the pocket still exhibits small fluctuations beyond 4 ns, suggesting that it retains some flexibility to accommodate the ligand's interactions. Hence, the RMSD values of the protein-ligand complexes were consistently low (below 3 Å), indicating that the compounds remained stably bound to Tau during the simulation. Furthermore, during the simulation, the system's total energy (H), potential energy (U), and kinetic energy (K), or the energy related to the motion of the atoms inside the system, varies within a specific range but stays comparatively constant overall (Figure S5).

RMSD Analysis of Tau with Drug 416 BRAF inhibitor/RG6344 Complex

In the earliest stages of the simulations, the ligand's RMSD fluctuates significantly, as it adapts to its ideal binding position inside the Tau protein's binding pocket (Figure 5a). These variations imply that the ligand is exploring several conformations in order to stabilize connections with critical residues. Following this phase, the ligand stabilizes, indicating that it has reached its

optimal binding site within the pocket. This stabilization is important because it confirms the ligand's effective interaction with the protein binding domain. Following this, the RMSD remained constant with just minimal changes, signifying that the ligand is currently securely fixed in the binding site and engaging with the Tau protein. The small departure from the initial structure indicates that the F-18 label does not disturb the ligand's conformation, facilitating proper attachment while still permitting PET scan imaging.

The Tau protein's RMSD is an estimation of how far it varies from its initial configuration when attached to the medication. In the initial 10 ns of the simulations, the protein fluctuates significantly as it experiences structural rearrangements to accept the F-18 tagged medication (Figure 5b). These oscillations are normal as the protein becomes stable in the drug-bound state. Following this adjustment time, the protein's RMSD lowers and stabilizes, suggesting that it has achieved a stable shape. This is significant because the protein has now established stable connections with the ligand, which is required for therapeutic efficacy. Beyond 10 ns, the protein's RMSD remains very steady, with only small changes, indicating that the Tau protein-ligand complex is generally stable.

The RMSD of the binding pocket changes throughout the simulation, indicating that the pocket has adapted to fit the ligand. At the beginning, the binding pocket displays significant RMSD fluctuations (Figure 5c), indicating the protein's flexibility as it underwent structural modifications to improve interactions with the medication. As the simulation advances, the binding pocket's RMSD grows, showing that the binding location has stabilized and adjusted to the ligand. Although minor changes persist, they indicate the pocket's preserved versatility, assuring the ligand is appropriately bound. Furthermore, the system's total energy (H), potential energy (U), and kinetic energy (K) are largely consistent within a specified range, demonstrating the system's overall stability (Figure S6).

RMSD Analysis of Tau with Drug 610 (Iloperidone/HP 873) Complex

The Tau/Drug 610 (Iloperidone/HP 873) complex's RMSD study offers important information about the interactions and structural stability of the Tau protein with the F-18-labeled medication, which has been tested for the therapeutic management of Alzheimer's disease. As seen in Figure 6a, the ligand first exhibits notable RMSD oscillations. The ligand adapts to occupy the Tau protein's binding pocket as best it can, this kind of action is to be predicted. The ligand's investigation of different conformations to determine the best stable binding location is reflected in these variations. Following the initial stages, the ligand's RMSD starts to regulate, suggesting that it has located its ideal location within the binding site and is engaging in stable interactions

with important residues. The ligand is now tightly attached, with few variations, indicating a robust and persistent binding throughout time. This stability contributes to the drug's efficacy as a medicinal agent. Additionally, the F-18 label does not appreciably change the ligand's structure, permitting consistent drug binding across the simulation.

The RMSD analysis of the Tau protein indicates its structural stability being attached to the F-18 labeled medication. The protein suffers considerable changes in the initial ten nanoseconds, indicating substantial structural modifications as it responds to drug interaction (Figure 6b). These modifications occur as the protein adjusts to its drug-bound structure. Following this modification, the protein's RMSD diminishes, indicating that drug binding has resulted in a stable configuration with stable connections between the ligand and protein required for the complex's stability. After 30 ns, the protein's RMSD is essentially steady, with just small changes, signifying a stable Tau-protein complex.

The RMSD of the binding pocket demonstrates the way the binding site adjusts to the drug. The pocket varies significantly during the beginning of the simulation (Figure 6c), indicating the binding site's versatility as it changes to fit the ligand. While the simulation continues, the RMSD of the binding pocket becomes stable, showing that the binding site has efficiently adjusted. This stabilization is critical for preserving the ligand's location and ensuring proper ligand-protein interactions. Across the simulation, the system's overall total energy, potential energy, and kinetic energy change along a narrow range while remaining relatively constant (Figure S7).

ADMET Analysis of the Top Target F-18 Labeled Drugs

The Fluorine-18 labeling of the selected compounds was successful, as indicated by the radiolabeling prediction. The Fluorine-18 isotope was attached to the fluorine-containing functional groups of the drugs, making them suitable for PET imaging. A potent lead chemical that targets a particular receptor should be quickly eliminated from the body and exhibit adequate intestinal absorption. The idea of drug-likeness acts as a crucial foundation for drug discovery. In pharmaceutical studies, Lipinski's Rule of Five (RO5) is frequently employed to assess pharmacokinetic features and drug-likeness of possible drug candidates. This phase assists in evaluating a compound's potential for oral bioavailability. A drug-like compound must meet Lipinski's criteria, which include having a molecular weight of less than 500, no more than five

hydrogen bond donors (HBDs), no more than ten HBAs, a logP of less than 5, and only one rule violation. In the present research, we used computational techniques to assess CBD's pharmacological characteristics, such as its ADMET profile, employing SwissADME http://www.swissadme.ch/ (Swiss Institute of Bioinformatics). The LogP values, which represent the partition coefficient between octanol and water (a measure of lipophilicity), range from 2.05 (Drug BRAF inhibitor/RG6344) to 2.72 (Drug Drug 610 (Iloperidone/HP 873)). LogP values around 2-3 are ideal for good membrane permeability, suggesting all three drugs are likely to have adequate bioavailability. The water solubility values are all positive, indicating higher solubility. Drug Drug 610 (Iloperidone/HP 873) has the highest water solubility (5.47), suggesting it may be more soluble than the others, which could potentially aid in faster absorption. All the three drugs are marked as having high gastrointestinal (GI) absorption, which means they are efficiently absorbed through the digestive tract. Further, each of the target drugs is classified as BBB permeant, indicating their ability to cross the blood-brain barrier, an essential feature for targeting the brain in Alzheimer's disease therapy. Drug BRAF inhibitor/RG6344 is not a P-glycoprotein substrate, which may reduce its efflux from cells, potentially enhancing its bioavailability. The other two drugs are substrates, which means they could be subject to active transport and removal from cells, potentially affecting their pharmacokinetics. Moreover, the 388 (BI-D1870) and Drug 610 (Iloperidone/HP 873) are inhibitors of several CYP enzymes, including CYP1A2, CYP2C19, CYP2C9, CYP2D6, and CYP3A4. Inhibition of these enzymes could alter the metabolism of other drugs, leading to potential drug-drug interactions. However, the BRAF inhibitor/RG6344 compound does not inhibit any of these enzymes, which may make it a safer option in terms of metabolic interactions. All three drugs have 0 PAINS (Pan Assay Interference Compounds) alerts, suggesting that none of them have properties likely to cause false positives in screening assays, an important consideration for their development as safe therapeutics. Lipinski's Rule of Five: All three drugs are Lipinski compliant, with having 0 violation. This suggests that they all possess favorable pharmacological properties for oral administration (Table 2). The ADMET analysis reveals that all three F-18 labeled drugs have favorable properties for targeting Alzheimer's disease. They exhibit good GI absorption, BBB permeability, and are unlikely to trigger significant toxicity, which is crucial for their application in the central nervous system. These findings are consistent with the goal of developing compounds that can effectively cross the blood-brain barrier and reach their target site in the brain.

Table 2. Physicochemical and pharmacokinetic properties of target drugs.

ADMET Properties	Drug 388 (BI-D1870)	Drug 416 BRAF inhibitor/RG6344	Drug Drug 610 (Iloperidone/HP 873)
Formula	C24H16F3N5	C20H17F2N5O4S	C24H27FN2O4
Molecular weight	428.42 g/mol	459.45 g/mol	425.48 g/mol
Num. heavy atoms	32	32	31
Fraction Csp3	0.08	0.25	0.42
Molar Refractivity	116.68	115.89	119.98
TPSA	77.05 ($Å^2$)	125.70 $Å^2$	64.80 ($Å^2$)
LogP	2.64	2.05	2.72
Water solubility	4.82	3.48	5.47
GI absorption (%)	High	High	High
BBB permeant	Yes	Yes	Yes
P-glycoprotein substrate	Yes	NO	Yes
CYP1A2 Inhibitor	Yes	NO	NO
CYP2C19 Inhibitor	Yes	NO	Yes
CYP2C9 Inhibitor	Yes	Yes	Yes
CYP2D6 Inhibitor	Yes	NO	Yes
CYP3A4 Inhibitor	Yes	NO	Yes
PAINS	0 alert	0 alert	0 alert
Lipinski	Yes; 0 violation	Yes; 0 violation	Yes; 0 violation
Synthetic accessibility	4.14	3.89	3.55

Comparative Evaluation of the Lead Selected Drugs for Tau in AD

Following computational investigations, binding associations, and ADMET evaluation, BI-D1870 (388) emerges as the most promising option for Tau-targeted imaging in AD with F-18 PET. BI-D1870 had the highest binding potential to Tau (ΔG = -8.79 kcal/mol) and low RMSD oscillations in molecular dynamics simulations, indicating consistent and robust binding. Its effective ADMET characteristics, such as a LogP of 2.64, molecular weight of 428.42 g/mol, higher gastrointestinal

absorption, high solubility (4.82), and robust blood-brain barrier (BBB) permeability, makes it an excellent option for PET imaging. Its F-18 labeling potential favors its diagnostics and therapeutic applicability in AD.

RG6344 (416), a BRAF inhibitors, has a significantly lower Tau binding affinity (ΔG = -7.91 kcal/mol), yet continues to interacts strongly with important Tau residues. Its ADMET characteristics, including a LogP of 2.05, substantial gastrointestinal absorption, and high BBB permeability, make it a viable candidate for AD imaging. However, its significantly lower Tau binding affinity than BI-D1870 restricts its diagnostic usefulness. Its pharmacokinetic features are satisfactory, having a LogP value of 2.72, indicating adequate BBB penetration. Having a molecular weight of 425.48 g/mol, Iloperidone also follows the Lipinski Rule of Five. It has the greatest solubility of the three, at 5.47, which could aid in quicker absorption. Furthermore, it has significant GI absorption and BBB permeability. On the other hand, its lesser Tau binding affinity as in comparison with BI-D1870 and RG6344 limits its overall importance for Tau-targeted AD detection. In summary, BI-D1870 stands out as the most significant candidate for Tau-targeted imaging in AD due to its superior binding affinity with Tau receptor. Moreover, BI-D1870 is the most attractive option for PET imaging and possible therapeutic intervention in AD, despite the fact that RG6344 and Iloperidone exhibit promise due to their lower binding affinities and marginally less favorable ADMET profiles.

Conclusion and Future Directions

The computational strategy employed in this study has successfully identified and optimized F-18 labeled drug candidates for Tau imaging and Alzheimer's diagnosis. The drugs BI-D1870 (388), RG6344 (416), and Iloperidone (610) have substantial Tau binding capacity with ΔG values of -8.79 kcal/mol, -7.91 kcal/mol, and -6.88 kcal/mol, accordingly, indicating their potential for Alzheimer's disease diagnostics. Notwithstanding their intriguing qualities, numerous restrictions remain, such as the absence of clinical trials for BI-D1870 and RG6344, limiting their immediate use in clinical applications. Additionally, although in-silico calculations and virtual screening of 977 CNS-penetrant medicines revealed potent Tau binders, the compounds' practical potency in imaging research and capacity to be transformed into therapeutic interventions have yet to be confirmed. Future approaches include additional in vivo confirmation of these F-18 labeled medicines, pharmacokinetic optimization, and clinical studies to evaluate their potential for

improved Alzheimer's early identification and diagnosis. Furthermore, increased screening of various CNS-penetrant compounds, as well as further improvement of drug design computational methods, can help to identify more potent candidates. Finally, the fabrication of these novel PET imaging substances offers the possibility of revolutionizing Alzheimer's diagnosis and opening new paths for targeted therapy approaches.

Data availability

All data and code generated in this study, including molecular simulation files produced with MOE (Molecular Operating Environment), Python scripts for F-18 radiolabeling prediction implemented using RDKit, structure-data files (SDFs) of BBB-permeable compounds, pharmacokinetic prediction models, and analysis notebooks for blood-brain barrier permeability assessment, are publicly available in the GitHub repository at https://github.com/Chong-ChengCSU/Blood-Brain-Barrier-Permeable-Drugs-for-F-18-Labeling Molecular dynamics simulations were performed using MOE 2022.02, while F-18 labeling workflows were coded in Python 3.10 with RDKit 2023.03.3 and supplementary cheminformatics libraries.

Ethics declarations

Competing interests

The authors declare no competing interests.

Author’s Contribution

P.T. and C.C. conceived the study and designed the methodology. P.T. operated the software tools. X.C. and Y.L. performed validation and formal analysis of the data. P.L. and J.W. conducted investigations and provided resources. L.Z. and B.T. prepared the original draft and created visualizations. All authors contributed to writing review and editing, and provided supervision. C.C. administered the project and acquired funding.

Funding Source

This work was supported by an active grant from the Changde Science and Technology Bureau (Technology R&D and Innovation Guidance Program; Grant/Award Number: *Changke*

Han [2022] No. 51) with project title *"Application of Modified Volume-Viscosity Swallow Test in Neurodegenerative Diseases"*.

References

1. Zhang, J., et al., Recent advances in Alzheimer's disease: Mechanisms, clinical trials and new drug development strategies. 2024. **9**(1): p. 211.
2. Porsteinsson, A.P., et al., Diagnosis of early Alzheimer's disease: clinical practice in 2021. 2021. **8**(3): p. 371-386.
3. Al-Zghool, O., DEVELOPMENT OF RADIOPHARMACEUTICALS FOR ALZHEIMER'S DISEASE.
4. Chen, S., et al., The global macroeconomic burden of Alzheimer's disease and other dementias: estimates and projections for 152 countries or territories. 2024. **12**(9): p. e1534-e1543.
5. Ye, J., et al., Targeting tau in Alzheimer's disease: from mechanisms to clinical therapy. 2024. **19**(7): p. 1489-1498.
6. Rabinovici, G.D., et al., Updated appropriate use criteria for amyloid and tau PET: A report from the Alzheimer's Association and Society for Nuclear Medicine and Molecular Imaging Workgroup. 2025. **21**(1): p. e14338.
7. Rawat, P., et al., Phosphorylated tau in Alzheimer's disease and other tauopathies. 2022. **23**(21): p. 12841.
8. Gonzalez-Ortiz, F., et al., Plasma brain-derived tau is an amyloid-associated neurodegeneration biomarker in Alzheimer's disease. 2024. **15**(1): p. 2908.
9. Lindberg, A., et al., Ligand-based design of [18F] OXD-2314 for PET imaging in non-Alzheimer's disease tauopathies. 2024. **15**(1): p. 5109.
10. Binmujlli, M.A.J.M., Exploring Radioiodinated Anastrozole and Epirubicin as AKT1-Targeted Radiopharmaceuticals in Breast Cancer: In Silico Analysis and Potential Therapeutic Effect with Functional Nuclear Imagining Implications. 2024. **29**(17): p. 4203.
11. Klyucherev, T.O., et al., Advances in the development of new biomarkers for Alzheimer's disease. 2022. **11**(1): p. 25.
12. Alzamil, Y., Advancements in PET and SPECT Imaging for Brain Neuron Receptors: Insights, Challenges, and Future Directions. 2025.
13. Gheisari, F. and R.J.M.J.o.C.R. Vali, Revolutionizing Diagnostic Precision: Emerging Trends and Innovations in Theranostics for Nuclear Medicine. 2024. **9**(2): p. 1-9.
14. Mukherjee, M.B., The Role of Neuro-Imaging Biomarkers in the Early Detection of Dementia and Alzheimer's Disease, in Exploring Medical Biotechnology-in vivo, in vitro, in silico. 2024, CRC Press. p. 112-118.
15. Wu, J., et al., Radiolabeled imaging agents for Alzheimer's disease. 2024. **2**(1): p. 80-95.
16. Wongso, H., R. Harada, and S.J.A.C.N. Furumoto, Current Progress and Future Directions in Non-Alzheimer's Disease Tau PET Tracers. 2025.
17. Lindberg, A., et al., Radiosynthesis, In Vitro Characterization, and In Vivo PET Neuroimaging of [18F] F-4 for Tau Protein: A First-in-Human PET Study. 2025. **16**(6): p. 1182-1189.
18. Tripathi, S.M., P. Chutia, and A.D. Murray, Neuroimaging Biomarkers in Alzheimer's disease. 2025.

19. Dasgupta, A., et al., Identification of novel brain penetrant GSK-3β inhibitors toward Alzheimer’s disease therapy by virtual screening, molecular docking, dynamic simulation, and MMPBSA analysis. 2024: p. 1-27.
20. Chong, J.R., et al., Blood-based high sensitivity measurements of beta-amyloid and phosphorylated tau as biomarkers of Alzheimer’s disease: a focused review on recent advances. 2021. **92**(11): p. 1231-1241.
21. Gupta, M., J. Feng, and G.J.M. Bhisetti, Experimental and Computational Methods to Assess Central Nervous System Penetration of Small Molecules. 2024. **29**(6): p. 1264.
22. Hansson, O., et al., Blood biomarkers for Alzheimer’s disease in clinical practice and trials. 2023. **3**(5): p. 506-519.
23. Gonzalez-Ortiz, F., et al., Plasma phospho-tau in Alzheimer’s disease: towards diagnostic and therapeutic trial applications. 2023. **18**(1): p. 18.
24. Ossenkoppele, R., R. van der Kant, and O.J.T.L.N. Hansson, Tau biomarkers in Alzheimer's disease: towards implementation in clinical practice and trials. 2022. **21**(8): p. 726-734.
25. Gonzalez-Ortiz, F., et al., Brain-derived tau: a novel blood-based biomarker for Alzheimer’s disease-type neurodegeneration. 2023. **146**(3): p. 1152-1165.
26. Delaby, C., C. Hirtz, and S.J.R.N. Lehmann, Overview of the blood biomarkers in Alzheimer's disease: Promises and challenges. 2023. **179**(3): p. 161-172.
27. Akkaya, D., et al., In vitro and in silico investigation of FDA-approved drugs to be repurposed against Alzheimer's disease. 2024. **85**(3): p. e22184.
28. Chennai, H.Y., et al., Identification of potent acetylcholinesterase inhibitors as new candidates for Alzheimer disease via virtual Screening, Molecular Docking, Dynamic Simulation, and Molecular Mechanics–Poisson–boltzmann surface area calculations. 2024. **29**(6): p. 1232.
29. Jangid, K., et al., Virtual screening and molecular dynamics simulation approach for the identification of potential multi-target directed ligands for the treatment of Alzheimer’s disease. 2024. **42**(1): p. 509-527.
30. Wibawa, T.H.A., et al. Molecular Docking Study of Pramipexole Derivatives as Radiopharmaceutical Candidates for Brain Imaging. in E3S Web of Conferences. 2024. EDP Sciences.
31. Wolfe, M., et al., Rational Approach to Optimizing Conformation-Switching Aptamers for Biosensing Applications. ACS sensors, 2024. **9**(2): p. 717-725.

Figure Click here to access/download;Figure;Figures.docx

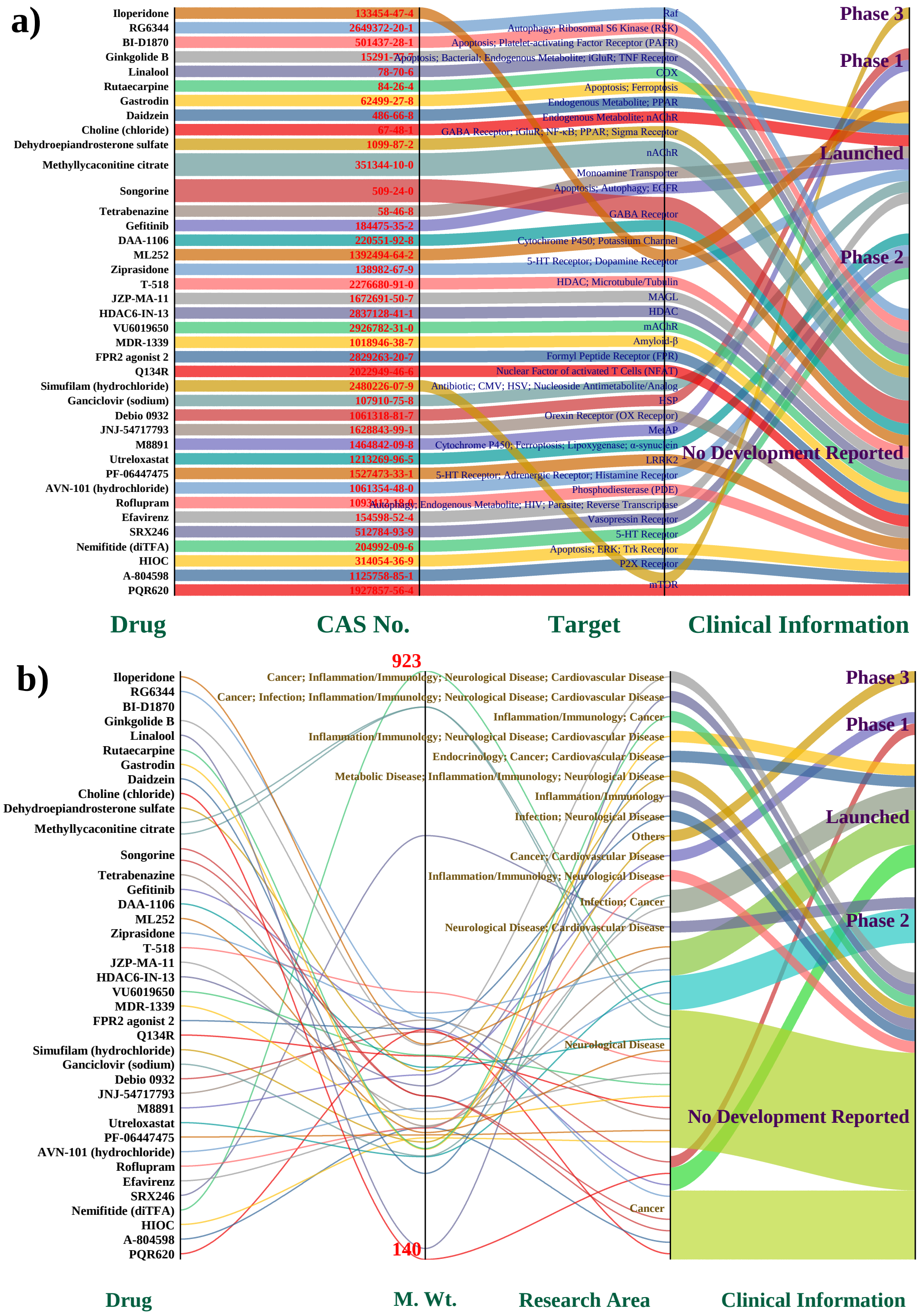

Figure 1. Visualization of the top 39 drugs selected after the first phase of virtual screening, based on binding free energies with tau, ADMET (Absorption, Distribution, Metabolism, Excretion, Toxicity), p-values, biological activities, and blood-brain barrier (BBB) permeabilities. (a) A Sankey diagram representing the progression of the selected drugs through different clinical phases (Phase 1, Phase 2, Phase 3, and launched). Each drug is shown with its respective CAS number and target, with the flow indicating its development status and phase. (b) A summary diagram highlighting the key characteristics of the selected drugs, including their molecular weights, research areas, clinical information, and the clinical development phases they are in.

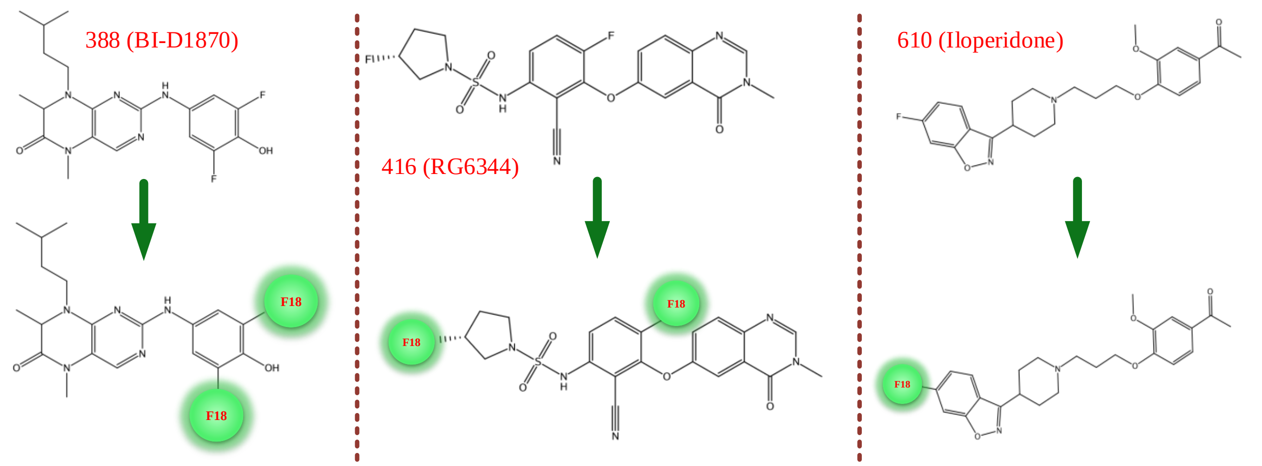

Figure 2: Structural representations of the top three compounds selected in the virtual screening process, shown both with and without the F-18 isotope label. The compounds 388 (BI-D1870), 416 (RG6344), and 610 (Iloperidone) are depicted in their standard forms (top) and with the F-18 isotope (bottom), highlighted in green circles.

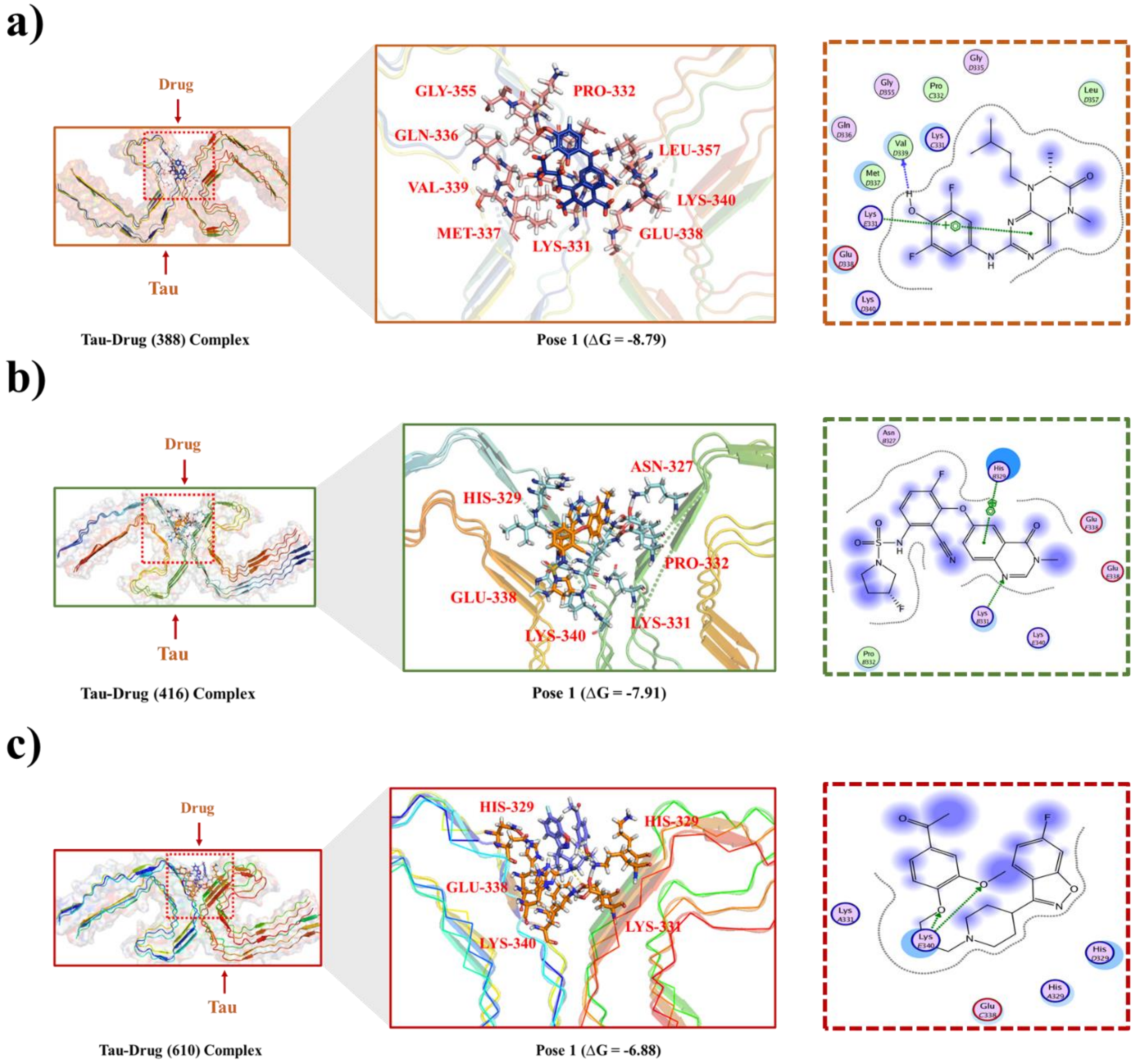

Figure 3. Interaction analysis of the Tau protein with the 388 (BI-D1870), BRAF inhibitor/RG6344 and Drug 610 (Drug 610 (Iloperidone/HP 873)) drugs complexes. **a)** 2D and 3D interaction diagrams of the docked complex of the drug 388 (BI-D1870) with Tau protein is presented. The left side displays closeup pictures of drug binding to Tau, proteins, highlighting hydrogen bonds and hydrophobic interactions. **b)** Drug (BRAF inhibitor/RG6344) interaction diagram in the active binding pocket of the Tau protein (both 2D and 3D). **c)** 2D and 3D interaction diagrams of Drug 610 (Drug 610 (Iloperidone/HP 873))-drug inside the Tau protein's active binding region.

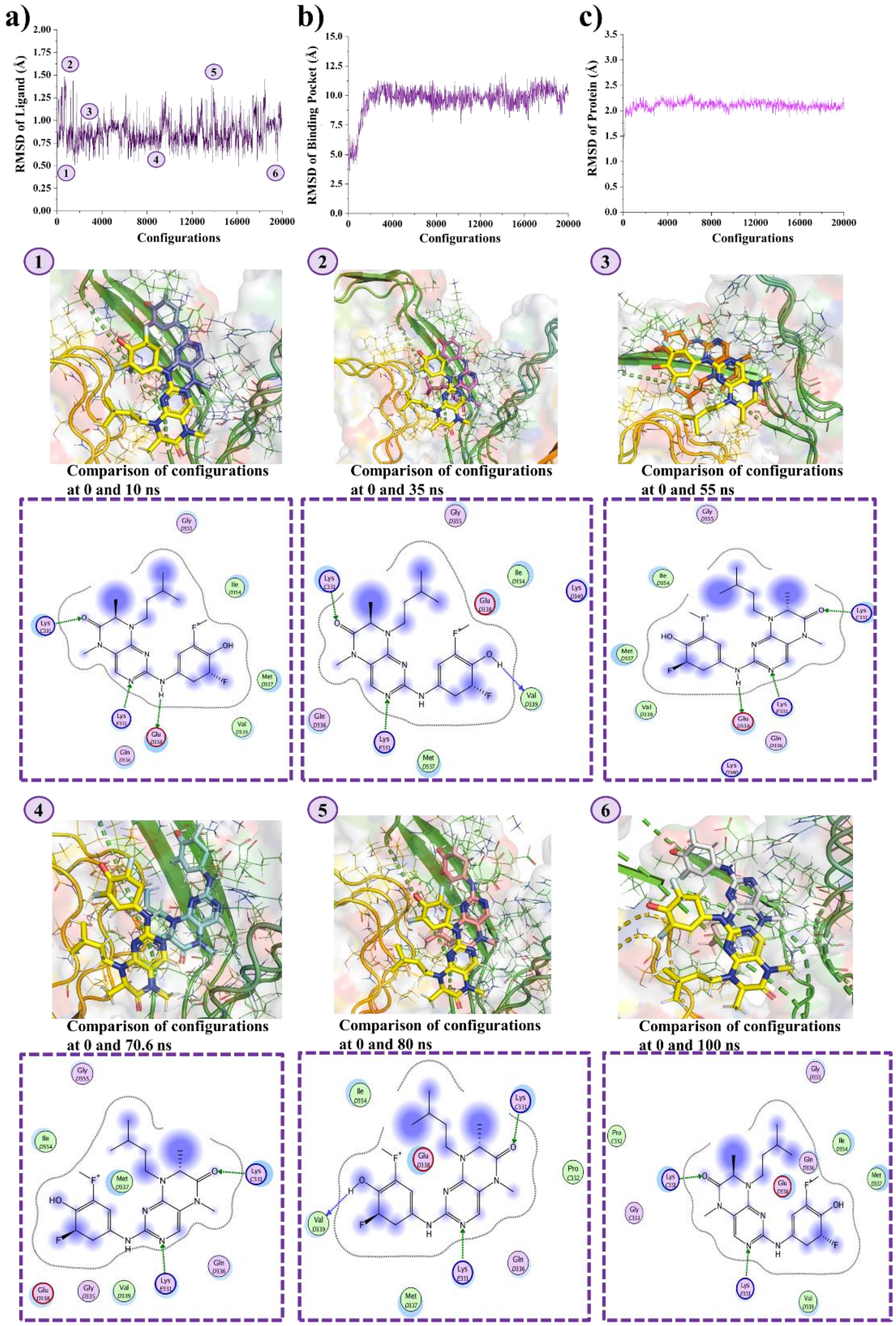

Figure 4. RMSD analysis of the 388 (BI-D1870)/Tau protein complex during a 100 ns. The integrity of the whole network is shown by **a)** the RMSD of drug (388 (BI-D1870)), **b)** the RMSD of the receptor protein, and **c)** the RMSD of the binding region. The small structural changes of the protein (1-6) demonstrate that binding of the target drug did not causes larger changes in the protein structure.

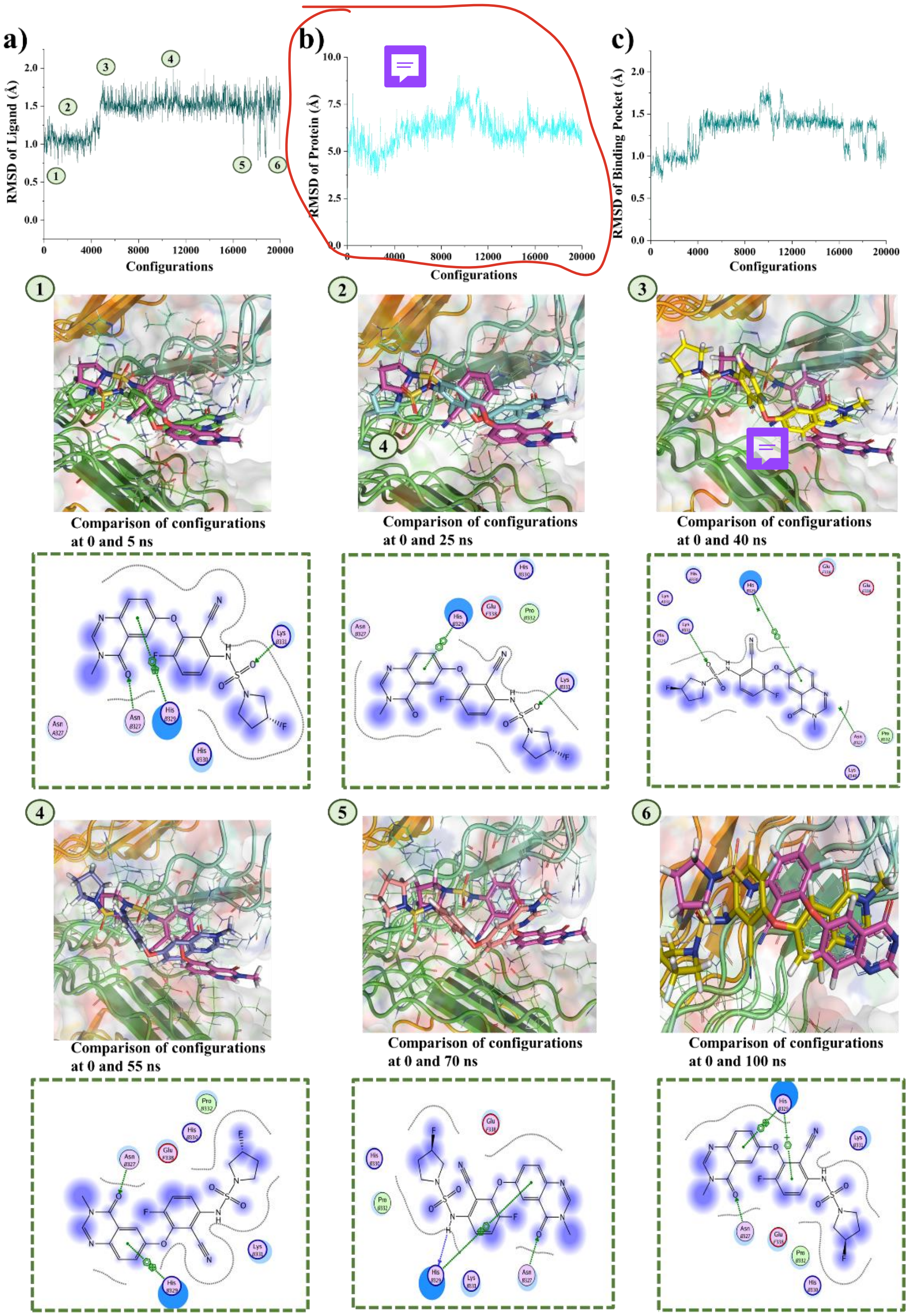

Figure 5. RMSD studies and the drug's interaction of the Tau protein with the F-18 labeled drug (BRAF inhibitor/RG6344). Key interactions between the target drug and the protein, including hydrogen bonding and hydrophobic interactions, are highlighted in the 2D interaction figures. **a)** the RMSD of drug (BRAF inhibitor/RG6344), **b)** the RMSD of the receptor protein, and **c)** the RMSD of the binding region. The RMSD analysis demonstrates the strong binding, which is essential for therapeutic use in Alzheimer's disease.

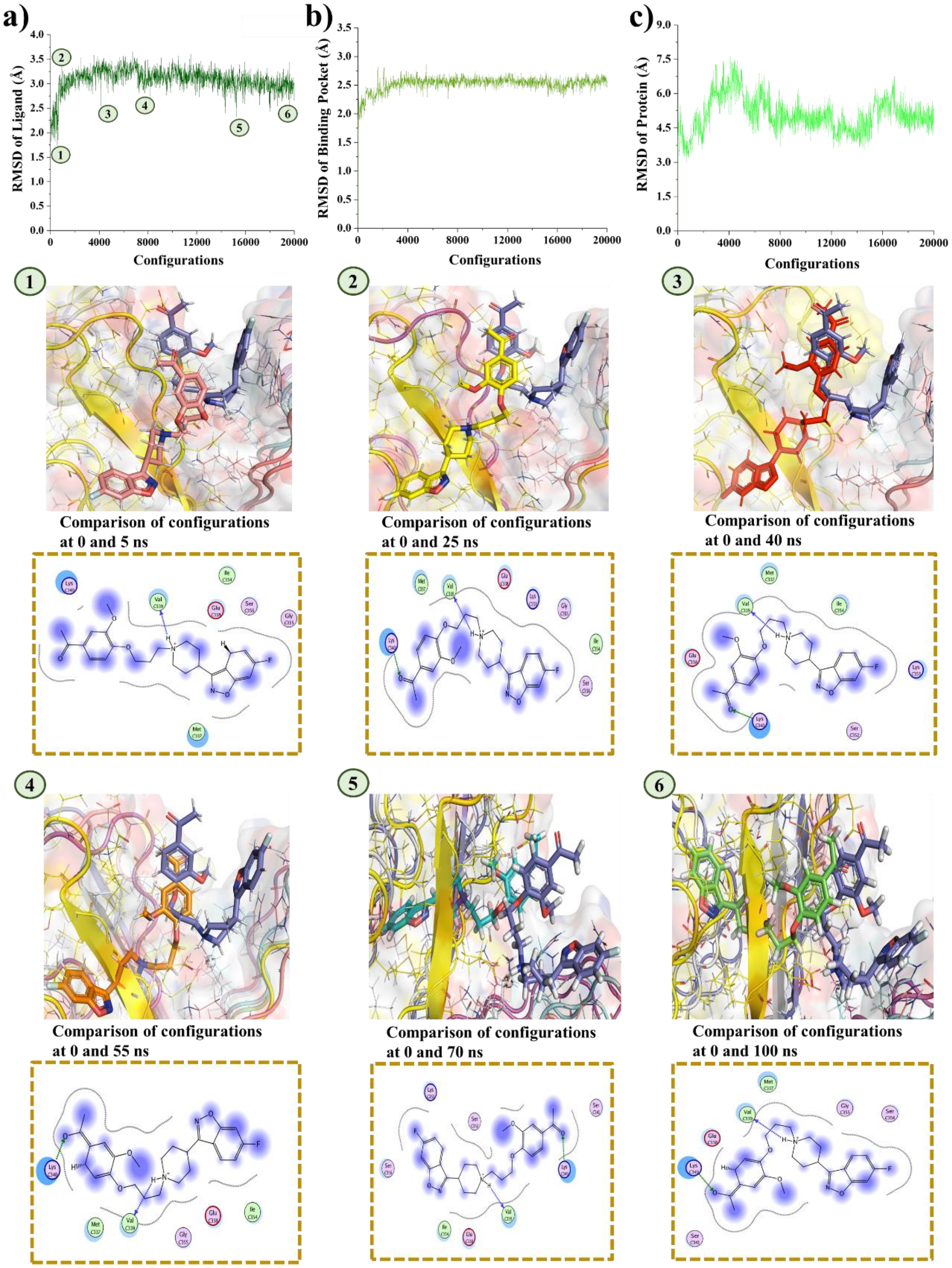

Figure 6. Molecular dynamics simulation analysis of the Tau protein with the target Drug 610 (Iloperidone/HP 873) drug (F-18 labeled). 2D interactions diagrams shows the hydrogen bonding and hydrophobic interactions between the drug and the receptor. **a)** the RMSD of drug (Drug 610 (Iloperidone/HP 873)), **b)** the RMSD of the receptor protein, and **c)** the RMSD of the binding region. The RMSD investigation validates the robust binding, which is crucial for the validation of the drug's stability within the Tau binding pocket.

Supporting Information

Click here to access/download
Supporting Information
SI.docx
