# Supplementary material for: Discovery of F-18 labeled repurposed CNS drugs by computational strategy for effective tau imaging and alzheimer’s diagnosis
Source: PLoS One. 2025 Dec 22;20(12):e0338901. doi: 10.1371/journal.pone.0338901 (PMC12721517; doi:10.1371/journal.pone.0338901)
Supplement: Attachment — Submitted filename: R1.+Response+Letter.docx [file pone.0338901.s014.docx]

Dear Dr. Gayathiri Ekambaram,

**Academic Editor**

PLOS One

Re: Submission of the Revised Manuscript with PONE-D-25-38504

## **Title: “Discovery of F-18 Labeled Repurposed CNS Drugs by Computational Strategy for Effective Tau Imaging and Alzheimer's Diagnosis”**

We are pleased to submit the revised manuscript as an article in *the PLOS One* for further consideration. We greatly appreciate the referees' positive comments, such as *“The research presented is of high quality and addresses a significant challenge in Alzheimer's diagnosis. The results are compelling, providing strong evidence for the effectiveness of the proposed computational strategy. The methodology is robust and the procedures are expertly executed, ensuring the reliability and validity of the findings. The manuscript is well-structured and clearly written, making the complex concepts accessible to a broader audience.* *The study is interesting and holds novelty in repurposing ligands as theradiagnostics.”* Moreover, we have carefully addressed the reviewers' concerns to make it even more suitable for the high standard of the *PLOS One*. A point-by-point response is appended, and changes to the main text are highlighted in yellow.

Thank you again for your time and efforts. We look forward to receiving updated comments. If you have any queries, please do not hesitate to contact me.

Sincerely,

Chong Cheng

On behalf of all co-authors.

**Additional Editor Comments:**

**Comment 1:** Can authors justify the 100 ns MD simulation duration as sufficiently robust for assessing Tau-drug complex stability? Are replicate simulations feasible?

**Response:** Thank you for your insightful comment. In response, we performed triplicate 100 ns MD simulations for each drug to assess the stability of the Tau-drug complex. This approach ensures the reliability and reproducibility of the results, and 100 ns is considered sufficient for observing meaningful equilibration and stability based on previous studies.

***MD Simulation Analysis***

*The docked complexes of the top three F-18 labeled drugs (388 (BI-D1870), BRAF inhibitor/RG6344, and Drug 610 (Iloperidone/HP 873)) with the Tau receptor underwent MD simulations to assess the stability of their protein-ligand complexes. The simulations were conducted for 100 ns, with the MD simulation being performed three times for each drug to ensure the reproducibility and reliability of the results. The RMSD analysis of the ligand, binding pocket, and protein in complex with the drug provides valuable insights into the structural stability and dynamics of the system throughout the simulation.*

***RMSD Analysis of Tau with Drug 388 (BI-D1870) Complex***

*The RMSD analysis of the Tau-388 (BI-D1870) complex shows that the ligand undergoes noticeable fluctuations in the first 0 to 10 ns (configuration 0 to 5000), indicating adjustments within the binding pocket (Figure 4a). From 10 to 30 ns (configuration 5000 to 10000), the RMSD stabilizes, with minor changes observed around 35 ns (configuration 15000). After 50 ns (configuration 20000), the ligand's RMSD remains consistent, confirming stable binding. The protein shows initial fluctuations between 0 and 20 ns (configuration 0 to 8000), stabilizing after 20 ns (configuration 8000), with slight deviations around 40 ns (configuration 16000). The protein structure becomes stable, reflecting the ligand's effect (Figure 4b). The binding pocket shows significant fluctuations between 0 to 10 ns (configuration 0 to 5000), stabilizing after 10 ns (configuration 5000), with minor fluctuations after 30 ns (configuration 12000), indicating that the pocket maintains a stable conformation (Figure 4c). The energy profile reveals that the total energy (H) decreases from 0 to 5000 configurations, stabilizing between -120 to -140 kcal/mol, potential energy (U) stabilizes around 500 kcal/mol, and kinetic energy (K) fluctuates around 5000*

**Figure 4.** RMSD analysis of the 388 (BI-D1870)/Tau protein complex during a 100 ns. The integrity of the whole network is shown by **a)** the RMSD of drug (388 (BI-D1870)), **b)** the RMSD of the receptor protein, and **c)** the RMSD of the binding region. The small structural changes of the protein (1-6) demonstrate that binding of the target drug did not causes larger changes in the protein structure.

*kcal/mol, showing minimal fluctuations and indicating a stable, energetically favorable protein-ligand complex (Figure S5a, b and c). Overall, the complex remains stable across all three replicates (R1, R2, and R3).*

***RMSD Analysis of Tau with Drug 416 BRAF inhibitor/RG6344 Complex***

*In the early stages of the simulation, the ligand’s RMSD shows significant fluctuations between 0 to 5 ns (configuration 0 to 2500) as it adjusts its position within the binding pocket. From 5 to 25 ns (configuration 2500 to 12500), the RMSD decreases and stabilizes, indicating that the ligand has found a stable binding pose (Figure 5a). By 55 ns (configuration 22000), the ligand’s RMSD remains steady, with minor fluctuations, suggesting it is firmly anchored. The F-18 label does not disturb the ligand's conformation, allowing for stable attachment and proper PET imaging. The protein’s RMSD fluctuates slightly from 0 to 25 ns (configuration 0 to 12500) as it adjusts to the ligand binding. After 25 ns (configuration 12500), the protein stabilizes, with minor fluctuations and a slight increase at 40 ns (configuration 16000), indicating conformational adjustments (Figure 5b). The binding pocket shows fluctuations from 0 to 25 ns (configuration 0 to 12500), reflecting its flexibility, then stabilizes after 25 ns (configuration 12500), with minor fluctuations continuing after 55 ns (configuration 22000) (Figure 5c). The total energy (H), potential energy (U), and kinetic energy (K) remain consistent within a specified range, confirming the system’s overall stability (Figure S6a, b and c).*

***RMSD Analysis of Tau with Drug 610 (Iloperidone/HP 873) Complex***

*The RMSD of the Tau/Drug 610 (Iloperidone/HP 873) complex shows significant fluctuations during 0 to 5 ns (configuration 0 to 2500) as the ligand adjusts within the binding pocket (Figure 6a). Between 5 to 25 ns (configuration 2500 to 12500), the RMSD stabilizes, indicating that the ligand has found a stable position, with minor fluctuations continuing until 55 ns (configuration 22000). This suggests the ligand is securely anchored and that the F-18 label does not affect its binding. The protein’s RMSD fluctuates from 0 to 25 ns (configuration 0 to 12500) as it adapts to the ligand, stabilizing after 25 ns (configuration 12500), with minor deviations observed around 40 ns (configuration 16000). The protein reaches a stable conformation thereafter (Figure 6b).*

**Figure 5.** RMSD studies and the drug's interaction of the Tau protein with the F-18 labeled drug (BRAF inhibitor/RG6344). Key interactions between the target drug and the protein, including hydrogen bonding and hydrophobic interactions, are highlighted in the 2D interaction figures. **a)** the RMSD of drug (BRAF inhibitor/RG6344), **b)** the RMSD of the receptor protein, and **c)** the RMSD of the binding region. The RMSD analysis demonstrates the strong binding, which is essential for therapeutic use in Alzheimer's disease.

**Figure 6.** Molecular dynamics simulation analysis of the Tau protein with the target Drug 610 (Iloperidone/HP 873) drug (F-18 labeled). 2D interactions diagrams shows the hydrogen bonding and hydrophobic interactions between the drug and the receptor. **a)** the RMSD of drug (Drug 610 (Iloperidone/HP 873)), **b)** the RMSD of the receptor protein, and **c)** the RMSD of the binding region. The RMSD investigation validates the robust binding, which is crucial for the validation of the drug's stability within the Tau binding pocket.

*The binding pocket shows notable fluctuations between 0 to 25 ns (configuration 0 to 12500), stabilizing by 25 ns (configuration 12500) and continuing with minor fluctuations beyond 55 ns (configuration 22000), indicating retained flexibility (Figure 6c). Throughout the simulation, total energy, potential energy, and kinetic energy fluctuate within a narrow range, confirming the system’s overall stability (Figure S7a, b and c).*

**Comment 2:** Were statistical methods applied to validate docking scores and MD simulation RMSD data reproducibility? If so, please provide details.

**Response:** Thank you for your valuable feedback and for raising this important question. We appreciate your attention to the statistical validation of the docking scores and MD simulation RMSD data. We have indeed applied statistical methods to validate the reproducibility of the MD simulation RMSD data. Details of these statistical methods are provided in the methods and results section in the revised manuscript and highlighted.

***Methods:***

*To validate the reproducibility of RMSD data from MD simulations across three drugs (318, 416, and 610), statistical analyses were performed on combined data from three independent simulation replicas per drug. The simulations for each drug spanned 0 to 20,000 configurations, with measurements every 10 units, resulting in approximately 2,200-2,215 data points per replica. The datasets included the Hamiltonian (H), representing the total system energy measured during MD using MOE 2009-2010, along with potential energy (U), kinetic energy (K), and RMSD values for the ligand, binding pocket (or alpha carbon for drug 318), and alpha carbon (AC). For drug 610, the solvent-accessible surface area (ASA) was also available but excluded from primary analyses. Descriptive statistics, such as mean, standard deviation (SD), and coefficient of variation (CV = SD/mean × 100%), were calculated for H and RMSD metrics at each time point, across entire simulations, and collectively across all drugs to assess central tendency and variability. Convergence was evaluated by computing running averages and SDs over a 100-time-point moving window to confirm stabilization. Inter-replica variability was quantified through pairwise differences and SDs between replicas per drug, with one-way ANOVA applied to test for significant differences in means (p > 0.05 indicating reproducibility) both within each drug and across datasets. Pearson correlation coefficients were computed for pairwise comparisons of H and RMSD trends across replicas and drugs to evaluate consistency. Time-averaged values were derived from the equilibrated phase (last 10% of each simulation) and compared using t-tests or ANOVA to confirm similarity across replicas and drugs. RMSD trends across drugs were compared by downsampling replica-averaged values to 1,000 ps intervals for visualization and further analysis. Significant ANOVA results (p < 0.05) prompted post-hoc Tukey's Honestly Significant Difference (HSD) tests to identify pairwise differences between drugs, using all equilibrated phase data points (n ≈ 660 per drug) and controlling the family-wise error rate at α = 0.05. All analyses were performed using Python libraries such as NumPy, SciPy, Pandas, and Statsmodels.*

***Results:***

The statistical analysis confirmed the high reproducibility of RMSD data across the three replicas for each drug, with low variability in the mean H values for drugs 318, 416, and 610. For the last 100 time points of each simulation, the mean H values were consistent, with drug 318 ranging from -103.33 to -104.89 kcal/mol, drug 416 from 173.0 to 173.5 kcal/mol, and drug 610 from 182.7 to 183.5 kcal/mol. RMSD values for the ligand ranged from 0.85 to 0.87 Å (CV 10-12%) for drug 318, 1.55 to 1.60 Å (CV 9-10%) for drug 416, and 2.90 to 3.00 Å (CV 6-7%) for drug 610 (Table S1). RMSD of the pocket ranged from 2.0 to 2.1 Å (CV ~5%) for drug 318, 1.30 to 1.32 Å (CV 5-6%) for drug 416, and 2.50 to 2.55 Å (CV 3-4%) for drug 610 (Table 2, Table S3). RMSD of the protein (Alpha carbon) was 10.0-10.8 Å (CV 5-7%) for drug 318, 6.0-6.2 Å (CV 6-7%) for drug 416, and 4.90-5.00 Å (CV 9-10%) for drug 610 (Table S3). Convergence was achieved after ~20,000 configurations, with minimal inter-replica differences (|H| < 5 kcal/mol), and ANOVA confirmed no significant differences for H, RMSD ligand, and RMSD pocket (p > 0.05). Significant differences were observed for RMSD alpha carbon (p ≈ 0.03–0.04). Tukey’s HSD test identified all pairwise differences as significant (p < 0.001), with drug 318 showing the lowest RMSD values across all metrics and drug 610 exhibiting the highest. Time-averaged RMSD values for the equilibrated phase were: ligand 318: 0.895 ± 0.143 Å, 416: 1.548 ± 0.122 Å, 610: 2.970 ± 0.153 Å; pocket 318: 2.094 ± 0.061 Å, 416: 1.189 ± 0.128 Å, 610: 2.582 ± 0.088 Å; Protein 318: 10.543 ± 0.569 Å, 416: 5.959 ± 0.528 Å, 610: 5.060 ± 0.464 Å (Table S4). These results affirm the reliability and robustness of the docking and MD simulations, with drug 318 showing the most stable ligand-protein interactions, drug 416 exhibiting intermediate stability, and drug 610 showing the greatest flexibility, particularly in the ligand and pocket. The statistical significance (p < 0.0001) and effect sizes (e.g., 2.076 Å ligand RMSD gap between 318 and 610) validate the differences in binding affinity and structural adaptability among the drugs, supporting their differential therapeutic potential.

**Table 2.** SD, CV, and p-Values for RMSD Metrics Across DrugsThe table below summarizes the standard deviation (SD), coefficient of variation (CV = SD/mean × 100%), and p-values for the equilibrated phase (last 10% of simulations). Means and SDs are replica-pooled (n ≈ 660 per drug). ANOVA p-values are overall across drugs per metric (from one-way ANOVA). Tukey HSD p-values are adjusted pairwise (all < 0.001, indicating significance for every comparison: 318 vs. 416, 318 vs. 610, 416 vs. 610).

| ***RMSD Metric*** | ***Drug*** | ***Mean (Å)*** | ***SD (Å)*** | ***CV (%)*** | ***ANOVA p-value*** | ***Tukey HSD p-values (all pairs)*** |
| --- | --- | --- | --- | --- | --- | --- |
| *Ligand* | *318* | *0.895* | *0.143* | *16.0* | *< 0.0001* | *< 0.001* |
|  | *416* | *1.548* | *0.122* | *7.9* |  | *< 0.001* |
|  | *610* | *2.970* | *0.153* | *5.2* |  | *< 0.001* |
| *Pocket* | *318* | *2.094* | *0.061* | *2.9* | *< 0.0001* | *< 0.001* |
|  | *416* | *1.189* | *0.128* | *10.8* |  | *< 0.001* |
|  | *610* | *2.582* | *0.088* | *3.4* |  | *< 0.001* |
| *Alpha Carbon* | *318* | *10.543* | *0.569* | *5.4* | *< 0.0001* | *< 0.001* |
|  | *416* | *5.959* | *0.528* | *8.9* |  | *< 0.001* |
|  | *610* | *5.060* | *0.464* | *9.2* |  | *< 0.001* |

**Table S1.** Comparisons RMSD Ligand. All pairs show significant differences, with RMSD increasing from drug 318 (tightest binding) to 610 (most flexible ligand).

| ***Group 1*** | ***Group 2*** | ***Mean Diff (Å)*** | ***Lower CI (Å)*** | ***Upper CI (Å)*** | ***p-adj*** | ***Reject H₀*** |
| --- | --- | --- | --- | --- | --- | --- |
| *318* | *416* | *0.653* | *0.636* | *0.671* | *<0.001* | *Yes* |
| *318* | *610* | *2.076* | *2.058* | *2.094* | *<0.001* | *Yes* |
| *416* | *610* | *1.422* | *1.405* | *1.440* | *<0.001* | *Yes* |

**Table S2.** RMSD Pocket. All pairs differ significantly, with drug 416 showing the most stable pocket, while 318 and 610 exhibit greater dynamics (610 > 318).

| ***Group 1*** | ***Group 2*** | ***Mean Diff (Å)*** | ***Lower CI (Å)*** | ***Upper CI (Å)*** | ***p-adj*** | ***Reject H₀*** |
| --- | --- | --- | --- | --- | --- | --- |
| *318* | *416* | *-0.905* | *-0.917* | *-0.892* | *<0.001* | *Yes* |
| *318* | *610* | *0.488* | *0.476* | *0.501* | *<0.001* | *Yes* |
| *416* | *610* | *1.393* | *1.380* | *1.405* | *<0.001* | *Yes* |

**Table S3.** RMSD of Protein. All pairs are significantly different, with drug 318 showing the most protein backbone flexibility, followed by 416 and 610 (most rigid).

| ***Group 1*** | ***Group 2*** | ***Mean Diff (Å)*** | ***Lower CI (Å)*** | ***Upper CI (Å)*** | ***p-adj*** | ***Reject H₀*** |
| --- | --- | --- | --- | --- | --- | --- |
|  |  |  |  |  |  |  |
| *318* | *416* | *-4.584* | *-4.650* | *-4.517* | *<0.001* | *Yes* |
| *318* | *610* | *-5.483* | *-5.550* | *-5.416* | *<0.001* | *Yes* |
| *416* | *610* | *-0.899* | *-0.965* | *-0.833* | *<0.001* | *Yes* |

**Table S4.** Time-Averaged RMSD Values (Equilibrated Phase, Å) These confirm the trends: Drug 318 has the lowest ligand RMSD but highest Protein RMSD; drug 610 shows the opposite pattern.

| ***RMSD Metric*** | ***Drug 318*** | ***Drug 416*** | ***Drug 610*** |
| --- | --- | --- | --- |
| *Ligand* | *0.895 ± 0.143* | *1.548 ± 0.122* | *2.970 ± 0.153* |
| *Binding Pocket* | *2.094 ± 0.061* | *1.189 ± 0.128* | *2.582 ± 0.088* |
| *Protein* | *10.543 ± 0.569* | *5.959 ± 0.528* | *5.060 ± 0.464* |

**Comment 3:** How was toxicity specifically assessed for the F-18 labeled compounds? Are there computational or experimental data available addressing radiolabeled ligand safety?

**Response:** Thank you for your valuable comment. The toxicity of the ligands, including after radiolabeling, was analyzed using the Protox3.0 webserver for toxicity predictions. While experimental data specifically addressing the safety of the radiolabeled compounds is not yet available, we plan to conduct in vivo studies in future work to validate these findings.

*According to the toxicity analysis, Drug 388 (BI-D1870) exhibits the lowest toxicity risk, with most toxicity endpoints, such as CYP enzyme interactions, neurotoxicity, and respiratory toxicity, predicted to be inactive, suggesting a minimal likelihood of toxicity. Drug 416 (BRAF inhibitor/RG6344) presents a more mixed toxicity profile, with endpoints like CYP2D6, CYP2C9 interactions, and respiratory toxicity marked as active, indicating a moderate risk of toxicity. The probability values align with this, indicating a moderate likelihood of toxicity, particularly in carcinogenicity and immunotoxicity. Drug 610 (Iloperidone/HP 873) shows the highest potential for toxicity, with active predictions for multiple endpoints, including CYP2D6, CYP2C9, CYP3A4 inhibition, neurotoxicity, mutagenicity, and cardiotoxicity, indicating a higher probability of toxicity. The probability values confirm that this drug carries the highest risk, particularly in CYP enzyme inhibition, neurotoxicity, and cardiotoxicity. In summary, Drug 388 is the safest in terms of toxicity, while Drugs 416 and 610 show some risks, with Drug 610 being of particular concern due to its higher toxicity predictions. The ADMET analysis reveals that all three F-18 labeled drugs have favorable properties for targeting Alzheimer's disease. They exhibit good GI absorption, BBB permeability, and with no PAINS alerts, which is crucial for their application in the central nervous system. These findings are consistent with the goal of developing compounds that can effectively cross the blood-brain barrier and reach their target site in the brain.*

**Figure 7.** Radar plots illustrating the predicted toxicity profiles for three drugs: (a) Drug 388 (BI-D1870), (b) Drug 416 (BRAF inhibitor/RG6344), and (c) Drug 610 (Iloperidone/HP 873). The plots display toxicity predictions across multiple pharmacological endpoints, with active toxicity predictions highlighted in red and inactive predictions in purple. The probability of toxicity is represented by a color gradient from blue to green. These plots assess the potential risks of various adverse effects, including CYP enzyme inhibition, neurotoxicity, and cardiotoxicity.

**Comment 4:** Please provide a more balanced and expanded explanation of PAINS filtering and other pharmacological screening criteria to match the detail level of Lipinski’s rule description.

**Response:** Thank you for your helpful comment. We have expanded the explanation of PAINS filtering and other pharmacological screening criteria to provide a more balanced and detailed description, matching the level of detail provided for Lipinski's rule.

***ADMET Analysis of the Top Target F-18 Labeled Drugs***

*The Fluorine-18 labeling of the selected compounds was successful, as indicated by the radiolabeling prediction. The Fluorine-18 isotope was attached to the fluorine-containing functional groups of the drugs, making them suitable for PET imaging. A potent lead chemical that targets a particular receptor should be quickly eliminated from the body and exhibit adequate intestinal absorption. The idea of drug-likeness acts as a crucial foundation for drug discovery. In pharmaceutical studies, Lipinski's Rule of Five (RO5) is frequently employed to assess pharmacokinetic features and drug-likeness, focusing on oral bioavailability. A drug-like compound must have a molecular weight under 500, no more than five hydrogen bond donors, ten acceptors, a logP under 5, and only one violation. In the present research, we used computational techniques to assess CBD's pharmacological characteristics, such as its ADMET profile. The LogP values range from 2.05 (BRAF inhibitor/RG6344) to 2.72 (Drug 610/Iloperidone), indicating ideal membrane permeability and good bioavailability. All drugs show positive water solubility, with Drug 610 having the highest (5.47), suggesting faster absorption. All three drugs exhibit high gastrointestinal absorption and are BBB permeant, essential for Alzheimer’s treatment. BRAF inhibitor/RG6344 is not a P-glycoprotein substrate, potentially enhancing its bioavailability, while the other two drugs are substrates, possibly affecting their pharmacokinetics. Drugs 388 (BI-D1870) and 610 (Iloperidone) inhibit several CYP enzymes, which may lead to drug-drug interactions, while BRAF inhibitor/RG6344 does not, making it a safer option for metabolic interactions. All three drugs are Lipinski compliant, with having 0 violation, suggests that they all possess favorable pharmacological properties for oral administration. In addition to Lipinski's Rule of Five, which primarily assesses oral bioavailability, we employed more stringent filters to prioritize compounds with a low risk of promiscuous activity and favorable CNS multiparameter optimization (MPO) profiles. Specifically, we implemented Pan-Assay Interference Compounds (PAINS) filtering to eliminate molecules containing substructures known to cause false-positive results in biological assays. These interfering motifs, such as certain enones, catechols, or rhodanines, can react non-specifically with protein targets or exhibit assay-dependent fluorescence, leading to misleading conclusions about true efficacy. The absence of PAINS alerts (0 alerts) for all three candidate drugs significantly increases confidence in their specific, target-driven binding to Tau. This suggests that these drugs are more likely to yield accurate results in biological assays, which is essential for their development as safe and effective therapeutic candidates.*

**Comment 5:** Confirm that all figures have been revised for proper labeling and alignment consistent with journal style.

**Response:** Thank you for your valuable comment. We have thoroughly reviewed all figures in the manuscript to ensure that the labeling and alignment are consistent with the journal's style guidelines. Any discrepancies have been corrected, and we have made sure that the figures are properly labeled and formatted to meet the required standards.

**Comment 6:** What are the authors’ plans for experimental validation (in vitro/in vivo) of these candidates? Please discuss limitations of the current computational-only approach.

**Response:** Thank you for your insightful comment. We agree that experimental validation is essential to confirm the findings from our computational approach.

*While this study primarily focuses on the in-silico predictions, we plan to conduct in vitro and in vivo experiments in future work to validate the interactions and efficacy of these candidates. These experimental studies will help assess the biological relevance of the computational results and provide further support for the potential therapeutic application of the candidates. We also acknowledge the limitations of a computational-only approach, notwithstanding their intriguing qualities, numerous restrictions remain, such as the absence of clinical trials for BI-D1870 and RG6344, limiting their immediate use in clinical applications. Additionally, although in-silico calculations and virtual screening of 977 CNS-penetrant medicines revealed potent Tau binders, the compounds' practical potency in imaging research and capacity to be transformed into therapeutic interventions have yet to be confirmed. Future approaches include additional in vivo confirmation of these F-18 labeled medicines, pharmacokinetic optimization, and clinical studies to evaluate their potential for improved Alzheimer's early identification and diagnosis.*

**Reviewer #1:**

**General Comments:**

My decision is to accept this manuscript, PONE-D-25-38504, for publication. The research presented is of high quality and addresses a significant challenge in Alzheimer's diagnosis. The results are compelling, providing strong evidence for the effectiveness of the proposed computational strategy. The methodology is robust and the procedures are expertly executed, ensuring the reliability and validity of the findings. The manuscript is well-structured and clearly written, making the complex concepts accessible to a broader audience. I have no major concerns regarding the research ethics or potential for dual publication. The authors have made a significant contribution to the field, and this work will be a valuable addition to the scientific literature. I commend the authors on their excellent work.

**Response:** We sincerely appreciate Reviewer positive feedback on our manuscript. We are grateful for the recognition of the high quality of our research, the significance of the challenge addressed in Alzheimer's diagnosis, and the effectiveness of our proposed computational strategy. We also value your acknowledgment of the robustness of our methodology and the clarity of our manuscript, as we aimed to ensure both reliability and accessibility of the complex concepts. Your confidence in the ethical considerations and the potential impact of our study is greatly appreciated, and we are honored by your commendation. Thank you for your thoughtful and constructive evaluation, which motivates us as we continue contributing to this important field.

**Reviewer #2:**

**General Comment:** The study is interesting and holds novelty in repurposing ligands as the radiagnostics. but the study has typos and needs language corrections. the method needs to be revised as well and needs to include more references.

**Response:** Thank you for your valuable feedback. We appreciate your recognition of the study's novelty in repurposing ligands as radiagnostics. We have corrected the typos and improved the language throughout the manuscript. Additionally, we revised the methodology for clarity and added more references to strengthen the context of our work. We believe these changes have enhanced the manuscript's quality.

**Comment 1:** MD simulation is run for only 100ns which is too short. the interpretation and discussion of MD also needs to be revised and rewritten.

**Response:** Thank you for your insightful comment. We have revised the interpretation and discussion of the MD simulation as per your suggestion. In addition to the 100 ns simulation, we performed triplicate MD simulations to ensure the reliability and reproducibility of our results. The revised manuscript now includes a more comprehensive analysis and clarification of the simulation duration and its relevance.

***MD Simulation Analysis***

*The docked complexes of the top three F-18 labeled drugs (388 (BI-D1870), BRAF inhibitor/RG6344 and Drug 610 (Iloperidone/HP 873) with Tau receptor underwent MD simulations to assess the stability of their protein-ligand complexes. The simulations were conducted for 100 ns, with the MD simulation being performed three times for each drug to ensure the reproducibility and reliability of the results. The RMSD analysis of the ligand, binding pocket, and protein in complex with the drug offers valuable insights into the structural stability and dynamics of the system throughout the MD simulation.*

***RMSD Analysis of Tau with Drug 388 (BI-D1870) Complex***

*The RMSD analysis of the Tau-388 (BI-D1870) complex shows that the ligand undergoes noticeable fluctuations in the first 0 to 10 ns (configuration 0 to 5000), indicating adjustments within the binding pocket (Figure 4a). From 10 to 30 ns (configuration 5000 to 10000), the RMSD stabilizes, with minor changes observed around 35 ns (configuration 15000). After 50 ns (configuration 20000), the ligand's RMSD remains consistent, confirming stable binding. The protein shows initial fluctuations between 0 and 20 ns (configuration 0 to 8000), stabilizing after 20 ns (configuration 8000), with slight deviations around 40 ns (configuration 16000). The protein structure becomes stable, reflecting the ligand's effect (Figure 4b). The binding pocket shows significant fluctuations between 0 to 10 ns (configuration 0 to 5000), stabilizing after 10 ns (configuration 5000), with minor fluctuations after 30 ns (configuration 12000), indicating that the pocket maintains a stable conformation (Figure 4c). The energy profile reveals that the total energy (H) decreases from 0 to 5000 configurations, stabilizing between -120 to -140 kcal/mol, potential energy (U) stabilizes around 500 kcal/mol, and kinetic energy (K) fluctuates around 5000 kcal/mol, showing minimal fluctuations and indicating a stable, energetically favorable protein-ligand complex (Figure S5a, b and c). Overall, the complex remains stable across all three replicates (R1, R2, and R3).*

***RMSD Analysis of Tau with Drug 416 BRAF inhibitor/RG6344 Complex***

*In the early stages of the simulation, the ligand’s RMSD shows significant fluctuations between 0 to 5 ns (configuration 0 to 2500) as it adjusts its position within the binding pocket. From 5 to 25 ns (configuration 2500 to 12500), the RMSD decreases and stabilizes, indicating that the ligand has found a stable binding pose (Figure 5a). By 55 ns (configuration 22000), the ligand’s RMSD remains steady, with minor fluctuations, suggesting it is firmly anchored. The F-18 label does not disturb the ligand's conformation, allowing for stable attachment and proper PET imaging. The protein’s RMSD fluctuates slightly from 0 to 25 ns (configuration 0 to 12500) as it adjusts to the ligand binding. After 25 ns (configuration 12500), the protein stabilizes, with minor fluctuations and a slight increase at 40 ns (configuration 16000), indicating conformational adjustments (Figure 5b).*

**Figure 4.** RMSD analysis of the 388 (BI-D1870)/Tau protein complex during a 100 ns. The integrity of the whole network is shown by **a)** the RMSD of drug (388 (BI-D1870)), **b)** the RMSD of the receptor protein, and **c)** the RMSD of the binding region. The small structural changes of the protein (1-6) demonstrate that binding of the target drug did not causes larger changes in the protein structure.

**Figure 5.** RMSD studies and the drug's interaction of the Tau protein with the F-18 labeled drug (BRAF inhibitor/RG6344). Key interactions between the target drug and the protein, including hydrogen bonding and hydrophobic interactions, are highlighted in the 2D interaction figures. **a)** the RMSD of drug (BRAF inhibitor/RG6344), **b)** the RMSD of the receptor protein, and **c)** the RMSD of the binding region. The RMSD analysis demonstrates the strong binding, which is essential for therapeutic use in Alzheimer's disease.

**Figure 6.** Molecular dynamics simulation analysis of the Tau protein with the target Drug 610 (Iloperidone/HP 873) drug (F-18 labeled). 2D interactions diagrams shows the hydrogen bonding and hydrophobic interactions between the drug and the receptor. **a)** the RMSD of drug (Drug 610 (Iloperidone/HP 873)), **b)** the RMSD of the receptor protein, and **c)** the RMSD of the binding region. The RMSD investigation validates the robust binding, which is crucial for the validation of the drug's stability within the Tau binding pocket.

*The binding pocket shows fluctuations from 0 to 25 ns (configuration 0 to 12500), reflecting its flexibility, then stabilizes after 25 ns (configuration 12500), with minor fluctuations continuing after 55 ns (configuration 22000) (Figure 5c). The total energy (H), potential energy (U), and kinetic energy (K) remain consistent within a specified range, confirming the system’s overall stability (Figure S6a, b and c).*

***RMSD Analysis of Tau with Drug 610 (Iloperidone/HP 873) Complex***

*The RMSD of the Tau/Drug 610 (Iloperidone/HP 873) complex shows significant fluctuations during 0 to 5 ns (configuration 0 to 2500) as the ligand adjusts within the binding pocket (Figure 6a). Between 5 to 25 ns (configuration 2500 to 12500), the RMSD stabilizes, indicating that the ligand has found a stable position, with minor fluctuations continuing until 55 ns (configuration 22000). This suggests the ligand is securely anchored and that the F-18 label does not affect its binding. The protein’s RMSD fluctuates from 0 to 25 ns (configuration 0 to 12500) as it adapts to the ligand, stabilizing after 25 ns (configuration 12500), with minor deviations observed around 40 ns (configuration 16000). The protein reaches a stable conformation thereafter (Figure 6b). The binding pocket shows notable fluctuations between 0 to 25 ns (configuration 0 to 12500), stabilizing by 25 ns (configuration 12500) and continuing with minor fluctuations beyond 55 ns (configuration 22000), indicating retained flexibility (Figure 6c). Throughout the simulation, total energy, potential energy, and kinetic energy fluctuate within a narrow range, confirming the system’s overall stability (Figure S7).*

*The statistical analysis confirmed the high reproducibility of RMSD data across the three replicas for each drug, with low variability in the mean H values for drugs 318, 416, and 610. For the last 100 time points of each simulation, the mean H values were consistent, with drug 318 ranging from -103.33 to -104.89 kcal/mol, drug 416 from 173.0 to 173.5 kcal/mol, and drug 610 from 182.7 to 183.5 kcal/mol. RMSD values for the ligand ranged from 0.85 to 0.87 Å (CV 10-12%) for drug 318, 1.55 to 1.60 Å (CV 9-10%) for drug 416, and 2.90 to 3.00 Å (CV 6-7%) for drug 610. RMSD of the pocket ranged from 2.0 to 2.1 Å (CV ~5%) for drug 318, 1.30 to 1.32 Å (CV 5-6%) for drug 416, and 2.50 to 2.55 Å (CV 3-4%) for drug 610. RMSD of the alpha carbon was 10.0-10.8 Å (CV 5-7%) for drug 318, 6.0-6.2 Å (CV 6-7%) for drug 416, and 4.90-5.00 Å (CV 9-10%) for drug 610. Convergence was achieved after ~20,000 configurations, with minimal inter-replica differences (|H| < 5 kcal/mol), and ANOVA confirmed no significant differences for H, RMSD ligand, and RMSD pocket (p > 0.05). Significant differences were observed for RMSD alpha carbon (p ≈ 0.03–0.04). Tukey’s HSD test identified all pairwise differences as significant (p < 0.001), with drug 318 showing the lowest RMSD values across all metrics and drug 610 exhibiting the highest. Time-averaged RMSD values for the equilibrated phase were: ligand 318: 0.895 ± 0.143 Å, 416: 1.548 ± 0.122 Å, 610: 2.970 ± 0.153 Å; pocket 318: 2.094 ± 0.061 Å, 416: 1.189 ± 0.128 Å, 610: 2.582 ± 0.088 Å; alpha carbon 318: 10.543 ± 0.569 Å, 416: 5.959 ± 0.528 Å, 610: 5.060 ± 0.464 Å. These results affirm the reliability and robustness of the docking and MD simulations, with drug 318 showing the most stable ligand-protein interactions, drug 416 exhibiting intermediate stability, and drug 610 showing the greatest flexibility, particularly in the ligand and pocket. The statistical significance (p < 0.0001) and effect sizes (e.g., 2.076 Å ligand RMSD gap between 318 and 610) validate the differences in binding affinity and structural adaptability among the drugs, supporting their differential therapeutic potential.*

**Comment 2:** The lipski's rule was explained in a lengthy way while PAINS was cut short and is not clear.

**Response:** Thank you for your thoughtful comment. We appreciate your feedback regarding the explanation of Lipinski's Rule and PAINS. In response, we have revised the manuscript to provide a more concise explanation of Lipinski's Rule and have expanded the section on PAINS to ensure clarity and sufficient detail.

***ADMET Analysis of the Top Target F-18 Labeled Drugs***

*The Fluorine-18 labeling of the selected compounds was successful, as indicated by the radiolabeling prediction. The Fluorine-18 isotope was attached to the fluorine-containing functional groups of the drugs, making them suitable for PET imaging. A potent lead chemical that targets a particular receptor should be quickly eliminated from the body and exhibit adequate intestinal absorption. The idea of drug-likeness acts as a crucial foundation for drug discovery. In pharmaceutical studies, Lipinski's Rule of Five (RO5) is frequently employed to assess pharmacokinetic features and drug-likeness, focusing on oral bioavailability. A drug-like compound must have a molecular weight under 500, no more than five hydrogen bond donors, ten acceptors, a logP under 5, and only one violation. In the present research, we used computational techniques to assess CBD's pharmacological characteristics, such as its ADMET profile. The LogP values range from 2.05 (BRAF inhibitor/RG6344) to 2.72 (Drug 610/Iloperidone), indicating ideal membrane permeability and good bioavailability. All drugs show positive water solubility, with Drug 610 having the highest (5.47), suggesting faster absorption. All three drugs exhibit high gastrointestinal absorption and are BBB permeant, essential for Alzheimer’s treatment. BRAF inhibitor/RG6344 is not a P-glycoprotein substrate, potentially enhancing its bioavailability, while the other two drugs are substrates, possibly affecting their pharmacokinetics. Drugs 388 (BI-D1870) and 610 (Iloperidone) inhibit several CYP enzymes, which may lead to drug-drug interactions, while BRAF inhibitor/RG6344 does not, making it a safer option for metabolic interactions. All three drugs are Lipinski compliant, with having 0 violation, suggests that they all possess favorable pharmacological properties for oral administration. In addition to Lipinski's Rule of Five, which primarily assesses oral bioavailability, we employed more stringent filters to prioritize compounds with a low risk of promiscuous activity and favorable CNS multiparameter optimization (MPO) profiles. Specifically, we implemented Pan-Assay Interference Compounds (PAINS) filtering to eliminate molecules containing substructures known to cause false-positive results in biological assays. These interfering motifs, such as certain enones, catechols, or rhodanines, can react non-specifically with protein targets or exhibit assay-dependent fluorescence, leading to misleading conclusions about true efficacy. The absence of PAINS alerts (0 alerts) for all three candidate drugs significantly increases confidence in their specific, target-driven binding to Tau. This suggests that these drugs are more likely to yield accurate results in biological assays, which is essential for their development as safe and effective therapeutic candidates.*

**Comment 3:** Please check the notes on the manuscript. the authors need to include more references, check their figures and align the labels (a,b,c).

**Response:** We appreciate your insightful comments and have addressed the points you raised. We have added additional references to further support the manuscript and have reviewed the figures to ensure proper alignment of the labels (a, b, c). These adjustments have been made and highlighted in the main manuscript to improve the clarity and accuracy.

**Comment 4:** Have the authors checked the toxicity of the ligands specifically after radiolabeling?

**Response:** Thank you for your valuable feedback and for raising an important consideration regarding the toxicity of the ligands after radiolabeling with Fluorine-18. The toxicity of the ligands, including after radiolabeling, was analyzed using the Protox3.0 webserver for toxicity predictions.

*According to the toxicity analysis, Drug 388 (BI-D1870) exhibits the lowest toxicity risk, with most toxicity endpoints, such as CYP enzyme interactions, neurotoxicity, and respiratory toxicity, predicted to be inactive, suggesting a minimal likelihood of toxicity. Drug 416 (BRAF inhibitor/RG6344) presents a more mixed toxicity profile, with endpoints like CYP2D6, CYP2C9 interactions, and respiratory toxicity marked as active, indicating a moderate risk of toxicity. The probability values align with this, indicating a moderate likelihood of toxicity, particularly in carcinogenicity and immunotoxicity. Drug 610 (Iloperidone/HP 873) shows the highest potential for toxicity, with active predictions for multiple endpoints, including CYP2D6, CYP2C9, CYP3A4 inhibition, neurotoxicity, mutagenicity, and cardiotoxicity, indicating a higher probability of toxicity.*

**Figure 7.** Radar plots illustrating the predicted toxicity profiles for three drugs: (a) Drug 388 (BI-D1870), (b) Drug 416 (BRAF inhibitor/RG6344), and (c) Drug 610 (Iloperidone/HP 873). The plots display toxicity predictions across multiple pharmacological endpoints, with active toxicity predictions highlighted in red and inactive predictions in purple. The probability of toxicity is represented by a color gradient from blue to green. These plots assess the potential risks of various adverse effects, including CYP enzyme inhibition, neurotoxicity, and cardiotoxicity.

*The probability values confirm that this drug carries the highest risk, particularly in CYP enzyme inhibition, neurotoxicity, and cardiotoxicity. In summary, Drug 388 is the safest in terms of toxicity, while Drugs 416 and 610 show some risks, with Drug 610 being of particular concern due to its higher toxicity predictions. The ADMET analysis reveals that all three F-18 labeled drugs have favorable properties for targeting Alzheimer's disease. They exhibit good GI absorption, BBB permeability, and with no PAINS alerts, which is crucial for their application in the central nervous system. These findings are consistent with the goal of developing compounds that can effectively cross the blood-brain barrier and reach their target site in the brain.*
