# Supplementary material for: Discovery of F-18 labeled repurposed CNS drugs by computational strategy for effective tau imaging and alzheimer’s diagnosis
Source: PLoS One. 2025 Dec 22;20(12):e0338901. doi: 10.1371/journal.pone.0338901 (PMC12721517; doi:10.1371/journal.pone.0338901)
Supplement: Attachment — Submitted filename: Final Response Letter for Editor+Reviewer.docx [file pone.0338901.s015.docx]

Dear Dr. Gayathiri Ekambaram,

**Academic Editor**

PLOS One

Re: Submission of the Revised Manuscript with PONE-D-25-38504

## **Title: “Discovery of F-18 Labeled Repurposed CNS Drugs by Computational Strategy for Effective Tau Imaging and Alzheimer's Diagnosis”**

We are pleased to submit the revised manuscript as an article in *the PLOS One* for further consideration. “Thank you for the positive feedback. We are grateful to the reviewers for their insightful evaluations, and we are pleased to hear that the revisions meet their expectations. We appreciate their time and effort in reviewing our manuscript, and we look forward to the next steps in the publication process.” Moreover, we have carefully addressed the reviewer concerns to make it even more suitable for the high standard of the *PLOS One*. A point-by-point response is appended, and changes to the main text are highlighted in yellow.

Thank you again for your time and efforts. We look forward to receiving updated comments. If you have any queries, please do not hesitate to contact me.

Sincerely,

Chong Cheng

On behalf of all co-authors.

**Additional Editor Comments:**

**Comment:** Based on the reviewers’ evaluations, I am pleased to inform you that your manuscript is scientifically sound and methodologically appropriate, and both reviewers acknowledged that you have addressed the earlier comments satisfactorily. However, Reviewer 3 (Dr. Abdallah Abdel Sattar) has raised a few minor concerns that should be addressed before final acceptance. Please carefully revise your manuscript.

**Response:** Thank you for your insightful comment. We sincerely appreciate the time and effort of you and the reviewers in evaluating our manuscript. We have carefully revised the manuscript in response to the reviewer 3 comments (Dr. Abdallah Abdel Sattar), addressing all concerns. We believe that these changes significantly improve the manuscript.

**Reviewer #3:**

**Comment 1:** Although the author made several modifications that enriched the work, the discussion is still weak, with an insufficient number of references in the results and discussion sections. So, I recommend editing the manuscript.

**Response:** We sincerely appreciate reviewer positive feedback on our manuscript. In response, we have revised the manuscript by adding relevant references to strengthen the discussion and better support the claims made (Highlighted in yellow in the main manuscript). These revisions enhance the scientific depth and alignment with current literature.

***Results and Discussion***

*The final retrieved and assembled library comprised 977 BBB-penetrant drug candidates with diverse mechanisms of action relevant to neurodegeneration and Tau pathology, including kinase inhibitors (e.g., CDK and mTOR inhibitors), autophagy regulators, and apoptosis inducers [1]. Notable examples include Flavopiridol (Phase 2, CDK inhibitor), PQR620 (mTOR inhibitor), and SNS-032 (CDK inhibitor, Phase 1). Prior evidence also indicates that CDK5 inhibition can reduce neurotoxicity in AD models [2-4]. Each compound was assessed for molecular weight, biological targets, and clinical phase data, alongside SMILES representations to facilitate in silico screening. Based on their pharmacological profile, known safety data, and chemical structures amenable to F-18 substitution, these compounds serve as a rationally designed set for docking studies and radiolabeling potential. The next step involved computational binding affinity evaluation with Tau protein, followed by selection of the top three candidates for F-18 labeling and PET imaging feasibility studies. Ensuring BBB permeability was essential for translational feasibility, as this property directly impacts the success of CNS-targeted radiopharmaceuticals [5]. Additionally, selecting molecules with F-18 labeling potential aligns with current trends in developing dual-purpose PET tracers that serve both diagnostic and therapeutic roles.*

***Virtual Screening Results for Tau***

*After conducting the virtual screening of 977 compounds using MOE, 39 compounds from the CNS-penetrant drug library were identified as strong binders to Tau (Figure 1). These compounds exhibited binding affinities with Tau ranging from -6.00 to -9.0 kcal/mol, demonstrating favorable interactions with the target protein. Specifically, the top 10 drug candidates had hydrophobic interactions and hydrogen bonds with the Tau microtubule-binding region, which is crucial for maintaining Tau's function and stability.* *These 39 compounds were successfully modeled with ^18^F atoms incorporated at viable positions, preserving chemical stability and pharmacophore integrity. Subsequent ADMET profiling further refined the selection of three compounds with molecular weights between 425-459 Da, within the acceptable drug-like range (350-520 Da), and demonstrated favorable solubility for intravenous delivery. Most importantly, logP values ranged from 2.05 to 2.72, indicating high BBB permeability, a critical parameter for neuroimaging agents. As visualized in Figure S1 a (clinical phase vs. research area) and e/f (molecular weight vs. clinical phase), these three candidates also aligned well with existing drug development trends and CNS-penetrant physicochemical space. Together, their strong Tau affinity, labeling feasibility, favorable ADMET properties, and CNS compatibility support their prioritization for future PET probe development in neurodegenerative disease research.*

*The virtual screening results demonstrate the robustness of the computational workflow in identifying potential Tau-binding ligands with docking energies comparable to clinically established tracers such as [¹⁸F]MK-6240 and [¹⁸F]PI-2620 [6, 7]. The presence of hydrophobic and hydrogen-bond interactions within the Tau microtubule-binding domain supports the likelihood of selective binding, a critical feature for imaging specificity. Additionally, the favorable logP range (2.0-3.0) and preserved pharmacophore integrity following F-18 modeling suggest that the top-ranked compounds possess the optimal balance of BBB permeability, solubility, and structural stability necessary for CNS imaging applications. These results collectively indicate that the identified candidates are promising scaffolds for developing next-generation Tau PET tracers with improved sensitivity and clinical translatability.*

**Figure 1.** Visualization of the top 39 drugs selected after the first phase of virtual screening, based on binding free energies with tau, ADMET (Absorption, Distribution, Metabolism, Excretion, Toxicity), p-values, biological activities, and blood-brain barrier (BBB) permeabilities. **(a)** A Sankey diagram representing the progression of the selected drugs through different clinical phases (Phase 1, Phase 2, Phase 3, and launched). Each drug is shown with its respective CAS number and target, with the flow indicating its development status and phase. **(b)** A summary diagram highlighting the key characteristics of the selected drugs, including their molecular weights, research areas, clinical information, and the clinical development phases they are in.

***Current Status of Selected Drugs***

*The selected drugs, 388 (BI-D1870), 416 (RG6344), and 610 (Iloperidone) presented in Figure 2, have shown promising potential in crossing the blood-brain barrier (BBB), making them significant candidates in neurological and cancer research. BI-D1870, an ATP-competitive inhibitor of ribosomal S6 kinase isoforms, has demonstrated potent activity with low nanomolar IC50 values for all RSK isoforms, indicating its potential as a therapeutic agent for autophagy and MAPK/ERK pathway-related disorders [8]. However, there is currently no development report available for this drug. RG6344, a potent BRAF inhibitor, is specifically designed for targeting BRAF V600-mutant solid tumors, such as colorectal cancer [9]. It is known for its activity in the MAPK/ERK pathway, but like BI-D1870, no clinical development has been reported so far. Iloperidone, an atypical antipsychotic drug, is already launched and used for the treatment of schizophrenia symptoms. It acts as a dual antagonist for both dopamine and serotonin receptors, contributing to its efficacy in neurological disorders [10, 11]. Despite their varied therapeutic targets, these drugs demonstrate effective blood-brain barrier penetration, which makes them valuable for further exploration in both cancer and neurological disease research. However, it is important to note that while Iloperidone has already been marketed, the other two drugs, BI-D1870 and RG6344, are still in the preclinical or early-stage development phase [12, 13]. The strong BBB permeability of BI-D1870, RG6344, and Iloperidone supports their potential as CNS imaging probes. BI-D1870’s modulation of the MAPK/ERK-RSK pathway is particularly relevant to Tau hyperphosphorylation and AD pathology. RG6344’s BRAF inhibition may similarly influence Tau-related signaling, while Iloperidone’s proven CNS safety profile enhances its translational viability. Collectively, these findings highlight their promise as repurposed candidates for Tau-targeted PET imaging.*

***Figure 2.*** *Structural representations of the top three compounds selected in the virtual screening process, shown both with and without the F-18 isotope label. The compounds 388 (BI-D1870), 416 (RG6344), and 610 (Iloperidone) are depicted in their standard forms (top) and with the F-18 isotope (bottom), highlighted in green circles.*

***Interaction Analysis of Drugs with Tau Protein***

*The F-18 labeled drug 388 (BI-D1870) interacts with several key amino acid residues of the Tau protein as shown in* ***Figure 3a****. The interaction diagram gives a detailed spatial view of how the drug interacts at the molecular level with the Tau protein structure, possibly inhibiting or modulating the Tau protein’s role in Alzheimer’s disease. The drug likely binds through hydrogen bond and hydrophobic interactions with the LEU-357, MET-337, PRO-332, GLN-336, GLU-338, LYS-340, GLY-355, LYS-331 and VAL-339, which prefer to interact with nonpolar groups crucial for the drug's action. The presence of alkyl or aromatic groups in the drug molecule likely facilitates these interactions that help stabilize the drug-protein complex. Further, the polar functional groups such as carbonyl or hydroxyl groups of the drug forms a hydrogen bond with LYS-331. The predicted binding free energy of the best three poses of drug-Tau complexes is ΔG = -8.79 kcal/mol, -8.10 kcal/mol and -7.85 kcal/mol respectively (Figure S2). The drug is shown to bind effectively to these residues, forming critical interactions that is key to the drug’s potential role in treating Alzheimer's disease, as it may disrupt Tau aggregation or improve brain function.*

**Figure 3.** Interaction analysis of the Tau protein with the 388 (BI-D1870), BRAF inhibitor/RG6344 and Drug 610 (Drug 610 (Iloperidone/HP 873)) drugs complexes. **a)** 2D and 3D interaction diagrams of the docked complex of the drug 388 (BI-D1870) with Tau protein is presented. The left side displays closeup pictures of drug binding to Tau, proteins, highlighting hydrogen bonds and hydrophobic interactions. **b)** Drug (BRAF inhibitor/RG6344) interaction diagram in the active binding pocket of the Tau protein (both 2D and 3D). **c)** 2D and 3D interaction diagrams of Drug 610 (Drug 610 (Iloperidone/HP 873))-drug inside the Tau protein's active binding region.

*The* ***Figure 3b*** *illustrates the binding between an F-18 labeled drug 416 (BRAF inhibitor/RG6344) and the Tau protein. The diagram shows that the drug interacts with several specific amino acid residues on the Tau protein, including* ***HIS-329****,* ***ASN-327****,* ***PRO-332****,* ***LYS-331****,* ***GLU-338*** *and* ***LYS-340****. These residues play a crucial role in the drug's effectiveness by forming various molecular interactions. Hydrophobic interactions are likely established between the drug and the nonpolar amino acid residues including* ***PRO-332, LYS-331, LYS-340, ASN-327*** *and* ***HIS-329****, helping to anchor the drug within the hydrophobic pocket of the Tau protein. Moreover, the* ***negatively charged GLU-338*** *residue may engage in* ***electrostatic interactions*** *with positively charged groups on the drug, further enhancing binding affinity.* ***Van der Waals forces*** *also contribute to the interaction, particularly with residues like* ***PRO-332.*** *The predicted binding free energy of the best three poses of drug-Tau complexes is ΔG = -7.91 kcal/mol, -7.73 kcal/mol and -7.25 kcal/mol respectively (Figure S3). The F-18 isotope attached to the drug 416-BRAF inhibitor/RG6344 plays a significant role in its detection, enabling the drug's distribution and interaction with Tau to be monitored through* ***PET*** *scans.*

*The binding interaction between F-18 labeled drug 610 (Iloperidone/HP 873) and the Tau protein is shown in Figure 3c. The Drug 610 (Iloperidone/HP 873) interacts with key residues in the Tau protein, including HIS-329, GLU-338, LYS-340, and LYS-331, each contributing to the drug's binding stability. HIS-329 and LYS-331 forms both hydrogen bonds with the drug's polar functional groups, anchoring it to the protein. Additionally, the carboxyl group of GLU-338 participates in electrostatic interactions with the drug's positively charged regions, enhancing the binding affinity. These interactions are crucial for the effective binding of the Drug 610 (Iloperidone/HP 873) to the Tau protein, suggesting its potential as a therapeutic agent for Alzheimer's disease. The predicted binding free energy of the best three poses of drug-Tau complexes is ΔG = -6.88 kcal/mol, -6.50 kcal/mol and -6.29 kcal/mol respectively (Figure S4). The docking score of all the complexes with CBD is presented in Table 1.*

**Table 1**. Docking score and interacting binding residues of Tau protein with different labelled drugs.

| *Drugs* | *Receptor* | *Complex*  *Poses* | *Binding Free Energy*  *(Kcal/mol)* | *Amino Acid Residues involved in binding interactions* |
| --- | --- | --- | --- | --- |
| *388 (BI-D1870)* | *Tau* | *Pose 1*  *Pose 2*  *Pose 3* | *-8.79*  *-8.10*  *-7.25* | *LEU-357, MET-337, PRO-332, GLN-336, GLU-338, LYS-340, GLY-355, LYS-331 and VAL-339* |
| *BRAF inhibitor/RG6344* | *-* | *Pose 1*  *Pose 2*  *Pose 3* | *-7.91*  *-7.73*  *-7.85* | *HIS-329, ASN-327, PRO-332, LYS-331, GLU-338 and LYS-340* |
| *Drug 610 (Drug 610 (Iloperidone/HP 873)* | *-* | *Pose 1*  *Pose 2*  *Pose 3* | *-6.88*  *-6.50*  *-6.29* | *HIS-329, GLU-338, LYS-340, and LYS-331* |

*(Pose 2 and 3 of all drugs are presented in S2, S3 and S4)*

*The interaction analysis highlights the strong and specific binding of all three compounds BI-D1870, RG6344, and Iloperidone to key residues within Tau’s microtubule-binding region. These residues (e.g., LYS-331, GLU-338, PRO-332) are critical for Tau aggregation, and their engagement suggests potential to interfere with fibril formation. The low binding free energies (-6.2 to -8.8 kcal/mol) indicate stable and high-affinity interactions comparable to reported Tau inhibitors [14, 15]. Among them, BI-D1870 exhibited the strongest interaction network and stability, reinforcing its suitability as a Tau-targeted radioligand. Overall, these findings suggest that F-18 labeling preserves molecular integrity while enabling visualization of Tau interactions, supporting their potential use as PET imaging probes in Alzheimer’s disease. Hence, the radiolabeled nature of the drug (F-18) enables its tracking through positron emission tomography (PET) imaging and monitoring the drug's distribution in the body, particularly in the brain, where Tau pathology is prominent. The radiolabeling with F-18 enhances the ability to visualize and track the drug's behavior in vivo, which is valuable for assessing its effectiveness in clinical settings. This combination of targeted drug binding and the diagnostic capability of F-18 labeling makes the drug an effective candidate for both treating Alzheimer's disease and monitoring treatment efficacy in clinical settings.*

***MD Simulation Analysis***

*The docked complexes of the top three F-18 labeled drugs (388 (BI-D1870), BRAF inhibitor/RG6344, and Drug 610 (Iloperidone/HP 873)) with the Tau receptor underwent MD simulations to assess the stability of their protein-ligand complexes. The simulations were conducted for 100 ns, with the MD simulation being performed three times for each drug to ensure the reproducibility and reliability of the results. The RMSD analysis of the ligand, binding pocket, and protein in complex with the drug provides valuable insights into the structural stability and dynamics of the system throughout the simulation.*

***RMSD Analysis of Tau with Drug 388 (BI-D1870) Complex***

*The RMSD analysis of the Tau-388 (BI-D1870) complex shows that the ligand undergoes noticeable fluctuations in the first 0 to 10 ns (configuration 0 to 5000), indicating adjustments within the binding pocket (Figure 4a). From 10 to 30 ns (configuration 5000 to 10000), the RMSD stabilizes, with minor changes observed around 35 ns (configuration 15000). After 50 ns (configuration 20000), the ligand's RMSD remains consistent, confirming stable binding. The protein shows initial fluctuations between 0 and 20 ns (configuration 0 to 8000), stabilizing after 20 ns (configuration 8000), with slight deviations around 40 ns (configuration 16000). The protein structure becomes stable, reflecting the ligand's effect (Figure 4b). The binding pocket shows significant fluctuations between 0 to 10 ns (configuration 0 to 5000), stabilizing after 10 ns (configuration 5000), with minor fluctuations after 30 ns (configuration 12000), indicating that the pocket maintains a stable conformation (Figure 4c). The energy profile reveals that the total energy (H) decreases from 0 to 5000 configurations, stabilizing between -120 to -140 kcal/mol, potential energy (U) stabilizes around 500 kcal/mol, and kinetic energy (K) fluctuates around 5000 kcal/mol, showing minimal fluctuations and indicating a stable, energetically favorable protein-ligand complex (Figure S5a, b and c). Overall, the complex remains stable across all three replicates (R1, R2, and R3).*

**Figure 4.** RMSD analysis of the 388 (BI-D1870)/Tau protein complex during a 100 ns. The integrity of the whole network is shown by **a)** the RMSD of drug (388 (BI-D1870)), **b)** the RMSD of the receptor protein, and **c)** the RMSD of the binding region. The small structural changes of the protein (1-6) demonstrate that binding of the target drug did not causes larger changes in the protein structure.

***RMSD Analysis of Tau with Drug 416 BRAF inhibitor/RG6344 Complex***

*In the early stages of the simulation, the ligand’s RMSD shows significant fluctuations between 0 to 5 ns (configuration 0 to 2500) as it adjusts its position within the binding pocket. From 5 to 25 ns (configuration 2500 to 12500), the RMSD decreases and stabilizes, indicating that the ligand has found a stable binding pose (Figure 5a). By 55 ns (configuration 22000), the ligand’s RMSD remains steady, with minor fluctuations, suggesting it is firmly anchored. The F-18 label does not disturb the ligand's conformation, allowing for stable attachment and proper PET imaging. The protein’s RMSD fluctuates slightly from 0 to 25 ns (configuration 0 to 12500) as it adjusts to the ligand binding. After 25 ns (configuration 12500), the protein stabilizes, with minor fluctuations and a slight increase at 40 ns (configuration 16000), indicating conformational adjustments (Figure 5b). The binding pocket shows fluctuations from 0 to 25 ns (configuration 0 to 12500), reflecting its flexibility, then stabilizes after 25 ns (configuration 12500), with minor fluctuations continuing after 55 ns (configuration 22000) (Figure 5c). The total energy (H), potential energy (U), and kinetic energy (K) remain consistent within a specified range, confirming the system’s overall stability (Figure S6a, b and c).*

**Figure 5.** RMSD studies and the drug's interaction of the Tau protein with the F-18 labeled drug (BRAF inhibitor/RG6344). Key interactions between the target drug and the protein, including hydrogen bonding and hydrophobic interactions, are highlighted in the 2D interaction figures. **a)** the RMSD of drug (BRAF inhibitor/RG6344), **b)** the RMSD of the receptor protein, and **c)** the RMSD of the binding region. The RMSD analysis demonstrates the strong binding, which is essential for therapeutic use in Alzheimer's disease.

***RMSD Analysis of Tau with Drug 610 (Iloperidone/HP 873) Complex***

*The RMSD of the Tau/Drug 610 (Iloperidone/HP 873) complex shows significant fluctuations during 0 to 5 ns (configuration 0 to 2500) as the ligand adjusts within the binding pocket (Figure 6a). Between 5 to 25 ns (configuration 2500 to 12500), the RMSD stabilizes, indicating that the ligand has found a stable position, with minor fluctuations continuing until 55 ns (configuration 22000). This suggests the ligand is securely anchored and that the F-18 label does not affect its binding. The protein’s RMSD fluctuates from 0 to 25 ns (configuration 0 to 12500) as it adapts to the ligand, stabilizing after 25 ns (configuration 12500), with minor deviations observed around 40 ns (configuration 16000). The protein reaches a stable conformation thereafter (Figure 6b). The binding pocket shows notable fluctuations between 0 to 25 ns (configuration 0 to 12500), stabilizing by 25 ns (configuration 12500) and continuing with minor fluctuations beyond 55 ns (configuration 22000), indicating retained flexibility (Figure 6c). Throughout the simulation, total energy, potential energy, and kinetic energy fluctuate within a narrow range, confirming the system’s overall stability (Figure S7).*

*The MD simulations confirmed the structural stability and strong binding of all three F-18-labeled drug-Tau complexes, with minimal RMSD fluctuations and consistent energy profiles, indicating thermodynamic equilibrium throughout the 100 ns trajectory. Among the three, BI-D1870 exhibited the lowest RMSD values and the most stable protein-ligand complex, suggesting robust and persistent binding interactions. These results are consistent with previous studies demonstrating that lower RMSD and energy convergence correlate with high ligand-receptor stability in Tau-targeted systems [15, 16]. The findings indicate that F-18 labeling does not destabilize ligand conformation or binding, supporting the feasibility of these compounds as reliable PET imaging probes for monitoring Tau aggregation in Alzheimer’s disease.*

**Figure 6.** Molecular dynamics simulation analysis of the Tau protein with the target Drug 610 (Iloperidone/HP 873) drug (F-18 labeled). 2D interactions diagrams shows the hydrogen bonding and hydrophobic interactions between the drug and the receptor. **a)** the RMSD of drug (Drug 610 (Iloperidone/HP 873)), **b)** the RMSD of the receptor protein, and **c)** the RMSD of the binding region. The RMSD investigation validates the robust binding, which is crucial for the validation of the drug's stability within the Tau binding pocket.

*The statistical analysis confirmed the high reproducibility of RMSD data across the three replicas for each drug, with low variability in the mean H values for drugs 318, 416, and 610. For the last 100 time points of each simulation, the mean H values were consistent, with drug 318 ranging from -103.33 to -104.89 kcal/mol, drug 416 from 173.0 to 173.5 kcal/mol, and drug 610 from 182.7 to 183.5 kcal/mol. RMSD values for the ligand ranged from 0.85 to 0.87 Å (CV 10-12%) for drug 318, 1.55 to 1.60 Å (CV 9-10%) for drug 416, and 2.90 to 3.00 Å (CV 6-7%) for drug 610 (Table S1). RMSD of the pocket ranged from 2.0 to 2.1 Å (CV ~5%) for drug 318, 1.30 to 1.32 Å (CV 5-6%) for drug 416, and 2.50 to 2.55 Å (CV 3-4%) for drug 610 (Table 2, Table S2). RMSD of the protein (Alpha carbon) was 10.0-10.8 Å (CV 5-7%) for drug 318, 6.0-6.2 Å (CV 6-7%) for drug 416, and 4.90-5.00 Å (CV 9-10%) for drug 610 (Table S3). Convergence was achieved after ~20,000 configurations, with minimal inter-replica differences (|H| < 5 kcal/mol), and ANOVA confirmed no significant differences for H, RMSD ligand, and RMSD pocket (p > 0.05). Significant differences were observed for RMSD alpha carbon (p ≈ 0.03–0.04). Tukey’s HSD test identified all pairwise differences as significant (p < 0.001), with drug 318 showing the lowest RMSD values across all metrics and drug 610 exhibiting the highest. Time-averaged RMSD values for the equilibrated phase were: ligand 318: 0.895 ± 0.143 Å, 416: 1.548 ± 0.122 Å, 610: 2.970 ± 0.153 Å; pocket 318: 2.094 ± 0.061 Å, 416: 1.189 ± 0.128 Å, 610: 2.582 ± 0.088 Å; Protein 318: 10.543 ± 0.569 Å, 416: 5.959 ± 0.528 Å, 610: 5.060 ± 0.464 Å (Table S4).*

**Table 2.** SD, CV, and p-Values for RMSD Metrics Across DrugsThe table below summarizes the standard deviation (SD), coefficient of variation (CV = SD/mean × 100%), and p-values for the equilibrated phase (last 10% of simulations). Means and SDs are replica-pooled (n ≈ 660 per drug). ANOVA p-values are overall across drugs per metric (from one-way ANOVA). Tukey HSD p-values are adjusted pairwise (all < 0.001, indicating significance for every comparison: 318 vs. 416, 318 vs. 610, 416 vs. 610).

| ***RMSD Metric*** | ***Drug*** | ***Mean (Å)*** | ***SD (Å)*** | ***CV (%)*** | ***ANOVA p-value*** | ***Tukey HSD p-values (all pairs)*** |
| --- | --- | --- | --- | --- | --- | --- |
| *Ligand* | *318* | *0.895* | *0.143* | *16.0* | *< 0.0001* | *< 0.001* |
|  | *416* | *1.548* | *0.122* | *7.9* |  | *< 0.001* |
|  | *610* | *2.970* | *0.153* | *5.2* |  | *< 0.001* |
| *Pocket* | *318* | *2.094* | *0.061* | *2.9* | *< 0.0001* | *< 0.001* |
|  | *416* | *1.189* | *0.128* | *10.8* |  | *< 0.001* |
|  | *610* | *2.582* | *0.088* | *3.4* |  | *< 0.001* |
| *Alpha Carbon* | *318* | *10.543* | *0.569* | *5.4* | *< 0.0001* | *< 0.001* |
|  | *416* | *5.959* | *0.528* | *8.9* |  | *< 0.001* |
|  | *610* | *5.060* | *0.464* | *9.2* |  | *< 0.001* |

*These results affirm the reliability and robustness of the docking and MD simulations, with drug 318 showing the most stable ligand-protein interactions, drug 416 exhibiting intermediate stability, and drug 610 showing the greatest flexibility, particularly in the ligand and pocket. The statistical significance (p < 0.0001) and effect sizes (e.g., 2.076 Å ligand RMSD gap between 318 and 610) validate the differences in binding affinity and structural adaptability among the drugs, supporting their differential therapeutic potential.*

***ADMET Analysis of the Top Target F-18 Labeled Drugs***

*The Fluorine-18 labeling of the selected compounds was successful, as indicated by the radiolabeling prediction. The Fluorine-18 isotope was attached to the fluorine-containing functional groups of the drugs, making them suitable for PET imaging. A potent lead chemical that targets a particular receptor should be quickly eliminated from the body and exhibit adequate intestinal absorption. The idea of drug-likeness acts as a crucial foundation for drug discovery. In pharmaceutical studies, Lipinski's Rule of Five (RO5) is frequently employed to assess pharmacokinetic features and drug-likeness, focusing on oral bioavailability. A drug-like compound must have a molecular weight under 500, no more than five hydrogen bond donors, ten acceptors, a logP under 5, and only one violation [17-19]. In the present research, we used computational techniques to assess CBD's pharmacological characteristics, such as its ADMET profile. The LogP values range from 2.05 (BRAF inhibitor/RG6344) to 2.72 (Drug 610/Iloperidone), indicating ideal membrane permeability and good bioavailability. All drugs show positive water solubility, with Drug 610 having the highest (5.47), suggesting faster absorption. All three drugs exhibit high gastrointestinal absorption and are BBB permeant, essential for Alzheimer’s treatment. BRAF inhibitor/RG6344 is not a P-glycoprotein substrate, potentially enhancing its bioavailability, while the other two drugs are substrates, possibly affecting their pharmacokinetics. Drugs 388 (BI-D1870) and 610 (Iloperidone) inhibit several CYP enzymes, which may lead to drug-drug interactions, while BRAF inhibitor/RG6344 does not, making it a safer option for metabolic interactions. All three drugs are Lipinski compliant, with having 0 violation, suggests that they all possess favorable pharmacological properties for oral administration. In addition to Lipinski's Rule of Five, which primarily assesses oral bioavailability, we employed more stringent filters to prioritize compounds with a low risk of promiscuous activity and favorable CNS multiparameter optimization (MPO) profiles. Specifically, we implemented Pan-Assay Interference Compounds (PAINS) filtering to eliminate molecules containing substructures known to cause false-positive results in biological assays. These interfering motifs, such as certain enones, catechols, or rhodanines, can react non-specifically with protein targets or exhibit assay-dependent fluorescence, leading to misleading conclusions about true efficacy. The absence of PAINS alerts (0 alerts) for all three candidate drugs significantly increases confidence in their specific, target-driven binding to Tau. This suggests that these drugs are more likely to yield accurate results in biological assays, which is essential for their development as safe and effective therapeutic candidates (Table 3).*

*According to the toxicity analysis, Drug 388 (BI-D1870) exhibits the lowest toxicity risk, with most toxicity endpoints, such as CYP enzyme interactions, neurotoxicity, and respiratory toxicity, predicted to be inactive, suggesting a minimal likelihood of toxicity. Drug 416 (BRAF inhibitor/RG6344) presents a more mixed toxicity profile, with endpoints like CYP2D6, CYP2C9 interactions, and respiratory toxicity marked as active, indicating a moderate risk of toxicity. The probability values align with this, indicating a moderate likelihood of toxicity, particularly in carcinogenicity and immunotoxicity. Drug 610 (Iloperidone/HP 873) shows the highest potential for toxicity, with active predictions for multiple endpoints, including CYP2D6, CYP2C9, CYP3A4 inhibition, neurotoxicity, mutagenicity, and cardiotoxicity, indicating a higher probability of toxicity (Figure 7).*

***Figure 7.*** *Radar plots illustrating the predicted toxicity profiles for three drugs: (a) Drug 388 (BI-D1870), (b) Drug 416 (BRAF inhibitor/RG6344), and (c) Drug 610 (Iloperidone/HP 873). The plots display toxicity predictions across multiple pharmacological endpoints, with active toxicity predictions highlighted in red and inactive predictions in purple. The probability of toxicity is represented by a color gradient from blue to green. These plots assess the potential risks of various adverse effects, including CYP enzyme inhibition, neurotoxicity, and cardiotoxicity.*

**Table 3.** Physicochemical and pharmacokinetic properties of target drugs.

| *ADMET Properties* | | *Drug*  *388 (BI-D1870)* | | *Drug 416*  *BRAF inhibitor/RG6344* | *Drug*  *Drug 610 (Iloperidone/HP 873)* |
| --- | --- | --- | --- | --- | --- |
| *Formula* | | | *C24H16F3N5* | *C20H17F2N5O4S* | *C24H27FN2O4* |
| *Molecular weight* | | | *428.42 g/mol* | *459.45 g/mol* | *425.48 g/mol* |
| *Num. heavy atoms* | | | *32* | *32* | *31* |
| *Fraction Csp3* | | | *0.08* | *0.25* | *0.42* |
| *Molar Refractivity* | | | *116.68* | *115.89* | *119.98* |
| *TPSA* | | | *77.05 (Å^2^)* | *125.70 Å²* | *64.80 (Å²)* |
| *LogP* | | | *2.64* | *2.05* | *2.72* |
| *Water solubility* | | | *4.82* | *3.48* | *5.47* |
|  | *GI absorption (%)* | | *High* | *High* | *High* |
| *BBB permeant* | | | *Yes* | *Yes* | *Yes* |
| *P-glycoprotein substrate* | | | *Yes* | *NO* | *Yes* |
| *CYP1A2 Inhibitor* | | | *Yes* | *NO* | *NO* |
| *CYP2C19 Inhibitor* | | | *Yes* | *NO* | *Yes* |
| *CYP2C9 Inhibitor* | | | *Yes* | *Yes* | *Yes* |
| *CYP2D6 Inhibitor* | | | *Yes* | *NO* | *Yes* |
| *CYP3A4 Inhibitor* | | | *Yes* | *NO* | *Yes* |
| *PAINS* | | | *0 alert* | *0 alert* | *0 alert* |
| *Lipinski* | | | *Yes;*  *0 violation* | *Yes; 0*  *violation* | *Yes; 0*  *violation* |
| *Synthetic accessibility* | | | *4.14* | *3.89* | *3.55* |

*The probability values confirm that this drug carries the highest risk, particularly in CYP enzyme inhibition, neurotoxicity, and cardiotoxicity. In summary, Drug 388 is the safest in terms of toxicity, while Drugs 416 and 610 show some risks, with Drug 610 being of particular concern due to its higher toxicity predictions. The ADMET analysis reveals that all three F-18 labeled drugs have favorable pharmacokinetic and physicochemical properties for targeting Alzheimer's disease. They exhibit good GI absorption, BBB permeability, and with no PAINS alerts, which is crucial for their application in the central nervous system. These findings are consistent with the goal of developing compounds that can effectively cross the blood-brain barrier and reach their target site in the brain. Further, the optimal LogP range (2.0-3.0), and full compliance with Lipinski’s rule of 5 indicate strong drug-likeness and efficient brain penetration. The absence of PAINS alerts enhances confidence in their target-specific activity, minimizing the risk of nonspecific interactions. Among the three, BI-D1870 exhibited the most balanced ADMET and lowest toxicity profile, suggesting a safer and more stable candidate for Tau PET probe development. RG6344 showed moderate metabolic stability, while Iloperidone’s higher CYP inhibition potential indicates a need for careful pharmacokinetic optimization. Overall, these findings highlight the translational potential of the identified compounds as radiolabeled imaging agents capable of effective brain targeting with acceptable safety margins [20].*

***Comparative Evaluation of the Lead Selected Drugs for Tau in AD***

*Following computational investigations, binding associations, and ADMET evaluation, BI-D1870 (388) emerges as the most promising option for Tau-targeted imaging in AD with F-18 PET. BI-D1870 had the highest binding potential to Tau (ΔG = -8.79 kcal/mol) and low RMSD oscillations in molecular dynamics simulations, indicating consistent and robust binding. Its effective ADMET characteristics, such as a LogP of 2.64, molecular weight of 428.42 g/mol, higher gastrointestinal absorption, high solubility (4.82), and robust blood-brain barrier (BBB) permeability, makes it an excellent option for PET imaging. Its F-18 labeling potential favors its diagnostics and therapeutic applicability in AD.*

*RG6344 (416), a BRAF inhibitors, has a significantly lower Tau binding affinity (ΔG = -7.91 kcal/mol), yet continues to interacts strongly with important Tau residues. Its ADMET characteristics, including a LogP of 2.05, substantial gastrointestinal absorption, and high BBB permeability, make it a viable candidate for AD imaging. However, its significantly lower Tau binding affinity than BI-D1870 restricts its diagnostic usefulness. Its pharmacokinetic features are satisfactory, having a LogP value of 2.72, indicating adequate BBB penetration. Having a molecular weight of 425.48 g/mol, Iloperidone also follows the Lipinski Rule of Five. It has the greatest solubility of the three, at 5.47, which could aid in quicker absorption. Furthermore, it has significant GI absorption and BBB permeability. On the other hand, its lesser Tau binding affinity as in comparison with BI-D1870 and RG6344 limits its overall importance for Tau-targeted AD detection. In summary, BI-D1870 stands out as the most significant candidate for Tau-targeted imaging in AD due to its superior binding affinity with Tau receptor. Moreover, BI-D1870 is the most attractive option for PET imaging and possible therapeutic intervention in AD, despite the fact that RG6344 and Iloperidone exhibit promise due to their lower binding affinities and marginally less favorable ADMET profiles. The comparative evaluation clearly establishes BI-D1870 as the most promising Tau-targeted PET candidate, owing to its highest binding affinity, stable molecular dynamics profile, and optimal ADMET characteristics. The strong and consistent Tau interaction pattern of BI-D1870 suggests its potential for high target selectivity and imaging contrast, similar to benchmark tracers such as [¹⁸F]PI-2620 and [¹⁸F]MK-6240 [6]. RG6344 demonstrated moderate stability and affinity, indicating potential as a secondary lead, while Iloperidone’s higher solubility but lower Tau binding limits its diagnostic efficiency. Collectively, these results confirm that BI-D1870 fulfills the key physicochemical and pharmacokinetic requirements for CNS PET tracers high binding affinity, low molecular weight, and optimal BBB permeability supporting its advancement for experimental validation in Alzheimer’s imaging studies.*

**Comment 2:** The docking binding free energies are too low and will not be selective for the tau protein; most likely, after performing docking to other proteins, it will give the same or maybe higher scores. So, the only study that used this protein needs to be cited as a reference: "doi: 10.1177/13872877251386440" to establish that the binding range for this protein is low.

**Response:** Thank you for your valuable feedback and for raising this important question.

*We have reviewed the suggested reference (DOI: 10.1177/13872877251386440) and incorporated it into our manuscript results section to address the concern regarding the low docking binding free energies and tau selectivity. This reference aligns with our findings, providing context for the observed low binding energies and reinforcing the challenges in achieving highly selective binding for tau.*

**Comment 3:** Why did the author select this mutated protein (9EOH) to do the docking process and no other crystal structures of tau?

**Response:** We appreciate your insightful comments and have addressed the points you raised.

*We selected the 9EOH mutated protein structure for the docking studies because it closely represents tau variants associated with Alzheimer's disease pathology. This specific mutation has been shown to promote tau aggregation, a critical feature in the development of neurodegenerative diseases such as Alzheimer's. While other tau crystal structures are available, 9EOH provides a more relevant model due to its disease-associated mutation, which aligns with the focus of our study on identifying drug candidates that interact with tau in its pathological form. Additionally, the 9EOH structure is well-characterized, with high-resolution data that allow for precise docking simulations. This made it an ideal choice for evaluating the binding affinities of F-18 labeled CNS drugs designed for tau imaging [21].*

***References***

*1.	Mueed, Z., et al., Tau and mTOR: The Hotspots for Multifarious Diseases in Alzheimer's Development. Frontiers in Neuroscience, 2019.* ***12****.*

*2.	Borsari, C., et al., 4-(Difluoromethyl)-5-(4-((3R,5S)-3,5-dimethylmorpholino)-6-((R)-3-methylmorpholino)-1,3,5-triazin-2-yl)pyridin-2-amine (PQR626), a Potent, Orally Available, and Brain-Penetrant mTOR Inhibitor for the Treatment of Neurological Disorders. Journal of medicinal chemistry, 2020.*

*3.	Jordà, E.G., et al., Neuroprotective action of flavopiridol, a cyclin-dependent kinase inhibitor, in colchicine-induced apoptosis. Neuropharmacology, 2003.* ***45****: p. 672-683.*

*4.	Pallàs, M., et al., Flavopiridol: an antitumor drug with potential application in the treatment of neurodegenerative diseases. Med Hypotheses, 2005.* ***64****(1): p. 120-3.*

*5.	Stanimirovic, D., et al., Blood–brain barrier models: in vitro to in vivo translation in preclinical development of CNS-targeting biotherapeutics. Expert Opinion on Drug Discovery, 2015.* ***10****: p. 141-155.*

*6.	Betthauser, T.J., et al., In Vivo Characterization and Quantification of Neurofibrillary Tau PET Radioligand (18)F-MK-6240 in Humans from Alzheimer Disease Dementia to Young Controls. J Nucl Med, 2019.* ***60****(1): p. 93-99.*

*7.	Mueller, A., et al., Tau PET imaging with (18)F-PI-2620 in Patients with Alzheimer Disease and Healthy Controls: A First-in-Humans Study. J Nucl Med, 2020.* ***61****(6): p. 911-919.*

*8.	Sapkota, G.P., et al., BI-D1870 is a specific inhibitor of the p90 RSK (ribosomal S6 kinase) isoforms in vitro and in vivo. Biochem J, 2007.* ***401****(1): p. 29-38.*

*9.	Sundar, R., et al., Targeting BRAF-Mutant Colorectal Cancer: Progress in Combination Strategies. Cancer Discov, 2017.* ***7****(6): p. 558-560.*

*10.	Kalkman, H.O., N. Subramanian, and D. Hoyer, Extended Radioligand Binding Profile of Iloperidone: A Broad Spectrum Dopamine/Serotonin/Norepinephrine Receptor Antagonist for the Management of Psychotic Disorders. Neuropsychopharmacology, 2001.* ***25****(6): p. 904-914.*

*11.	Tonin, F.S., et al., Iloperidone in the treatment of schizophrenia: an evidence-based review of its place in therapy. Core Evid, 2016.* ***11****: p. 49-61.*

*12.	Fontana, E., et al., Preliminary safety, pharmacokinetics, and clinical activity of RG6344 in patients with BRAF V600E-mutant metastatic colorectal cancer (mCRC). Journal of Clinical Oncology, 2025.* ***43****(16_suppl): p. 3542-3542.*

*13.	Derman, C., FDA Approves Iloperidone (Fanapt) for Acute Treatment of Bipolar I Disorder.*

*14.	Bi, X., et al., Exploring multitarget molecular mechanisms of cannabidiol in Alzheimer's disease treatment using molecular simulations and modeling. J Alzheimers Dis, 2025: p. 13872877251386440.*

*15.	Job, N., V.S. Thimmakondu, and K. Thirumoorthy, In Silico Drug Design and Analysis of Dual Amyloid-Beta and Tau Protein-Aggregation Inhibitors for Alzheimer’s Disease Treatment. Molecules, 2023.* ***28****(3): p. 1388.*

*16.	Chai, K., et al., Molecular Deformation Is a Key Factor in Screening Aggregation Inhibitor for Intrinsically Disordered Protein Tau. ACS Cent Sci, 2024.* ***10****(3): p. 717-728.*

*17.	Daoud, N.E.-H., et al., ADMET Profiling in Drug Discovery and Development: Perspectives of In Silico, In Vitro and Integrated Approaches. Current Drug Metabolism, 2021.* ***22****.*

*18.	Wu, F., et al., Computational Approaches in Preclinical Studies on Drug Discovery and Development. Front Chem, 2020.* ***8****: p. 726.*

*19.	Ajayi, E.D., et al., Breaking barriers: Medicinal chemistry strategies and advanced in-silico approaches for overcoming the BBB and enhancing CNS penetration. European Journal of Medicinal Chemistry, 2026.* ***301****: p. 118219.*

*20.	Zhang, J., et al., Computational toxicology in drug discovery: applications of artificial intelligence in ADMET and toxicity prediction. Briefings in Bioinformatics, 2025.* ***26****(5): p. bbaf533.*

*21.	Seidler, P.M., et al., Structure-based discovery of small molecules that disaggregate Alzheimer’s disease tissue derived tau fibrils in vitro. Nature Communications, 2022.* ***13****(1): p. 5451.*
